# Supplementary material for: A lentiviral vector B cell gene therapy platform for the delivery of the anti-HIV-1 eCD4-Ig-knob-in-hole-reversed immunoadhesin
Source: Mol Ther Methods Clin Dev. 2023 Feb 11;28:366–84. doi: 10.1016/j.omtm.2023.02.004 (PMC9984920; doi:10.1016/j.omtm.2023.02.004)
Supplement: Document S1. Figures S1–S5 [file mmc1.pdf]

OMTM, Volume *28*

Supplemental information

A lentiviral vector B cell gene therapy platform for the delivery of the anti-HIV-1 eCD4-Ig-knob-in-hole-reversed immunoadhesin

Eirini Vamva, Stosh Ozog, Daniel P. Leaman, Rene Yu-Hong Cheng, Nicholas J. Irons, Andee Ott, Claire Stoffers, Iram Khan, Geraldine K.E. Goebrecht, Matthew R. Gardner, Michael Farzan, David J. Rawlings, Michael B. Zwick, Richard G. James, and Bruce E. Torbett

Supplemental Figures

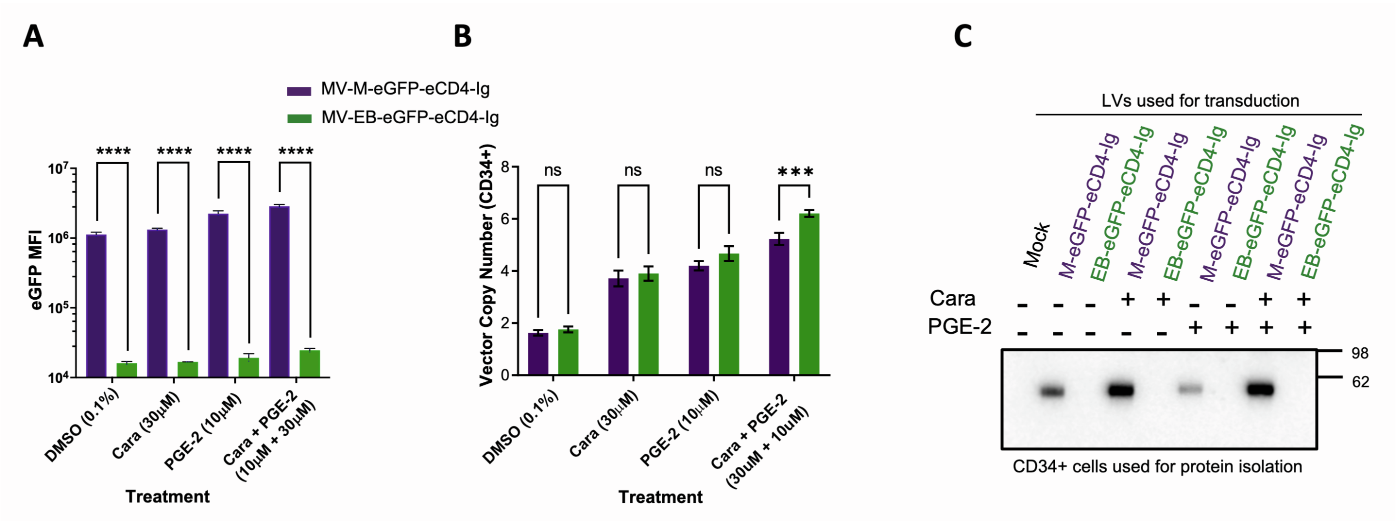

Figure S1. The B cell EµB29 promoter maintains low levels of eGFP expression in CD34^{+} stem and progenitors without detectable eCD4-Ig in the culture supernatant as LV copy number increases. [A] Small molecule enhancement of LV M-eGFP-eCD4-Ig or EB-eGFP-eCD4-Ig transduction of umbilical cord blood CD34^{+} stem and progenitors (HSPCs) and eGFP MFI detection 7 days post LV transduction. DMSO was the diluent for Cara and PGE-2. [B] Viral Copy Numbers (VCNs) from CD34^{+} HSPCs evaluated in [A] for eGFP expression. [C] Assessment of eCD4-Ig production in culture supernatants as in Figure 1 [D]. Data presented in bar graphs are mean ± S.D. of triplicates. ns = not significant, ***p<0.001, ****p<0.0001.

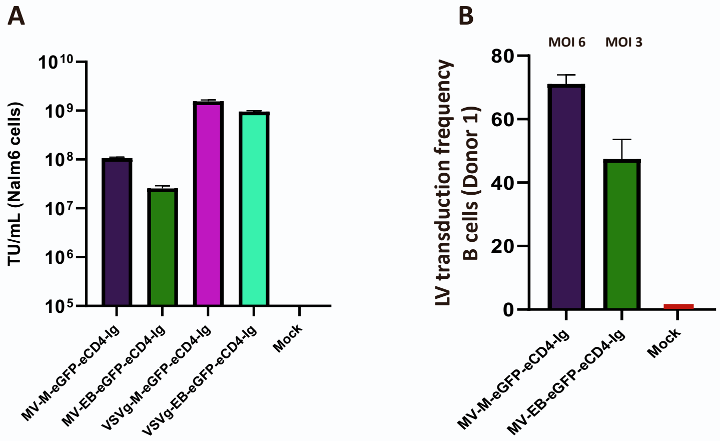

Figure S2. Measles pseudotyped (MV) and VSV-G LV transduction of Nalm6 B cells and MV LV transduction of primary B cells. [A] Titration of MV-M-eGFP-eCD4-Ig, MV-EB-eGFP-eCD4-Ig, VSV-G-M-eGFP-eCD4-Ig and VSV-G-MV-EB-eGFP-eCD4-Ig LVs by transduction of Nalm6 cells. Transduction units (TUs) were measured using flow cytometry five days post transduction (see Material and Methods for protocols). Transduction Units/mL (TU/mL) were calculated by flow cytometry of eGFP-expressing cells, using the dilution in which samples contained 4%–25% eGFP^{+} cells. **[B]** 20 µL of MV-M-eGFP-eCD4-Ig (MOI 6) and MV-EB-eGFP-eCD4-Ig (MOI 3) were used to transduce primary B cells (Donor 1) as described in Materials and Methods. Transduction efficiency was measured by evaluating primary B cells for eGFP production using flow cytometry five days post LV transduction. Mock LV used the same conditions as LV transduction, but without the addition of LV. B cell Donor 1.

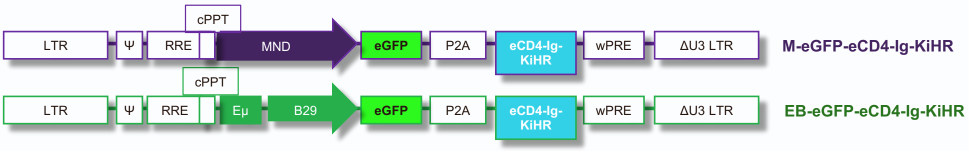

Figure S3. Diagram of LVs MND-eCD4-Ig-KiHR and EμB29-eCD4-Ig-KiHR. The diagram shows the inclusion of KiHR in eCD4-Ig (eCD4-Ig-KiHR) which includes the KiHR mutations F405A and T394F in the CH3 dimerization interface. See **Figure 3** for additional information on the CH3 mutations. Other LV elements remain the same as shown in **Figure 1B**.

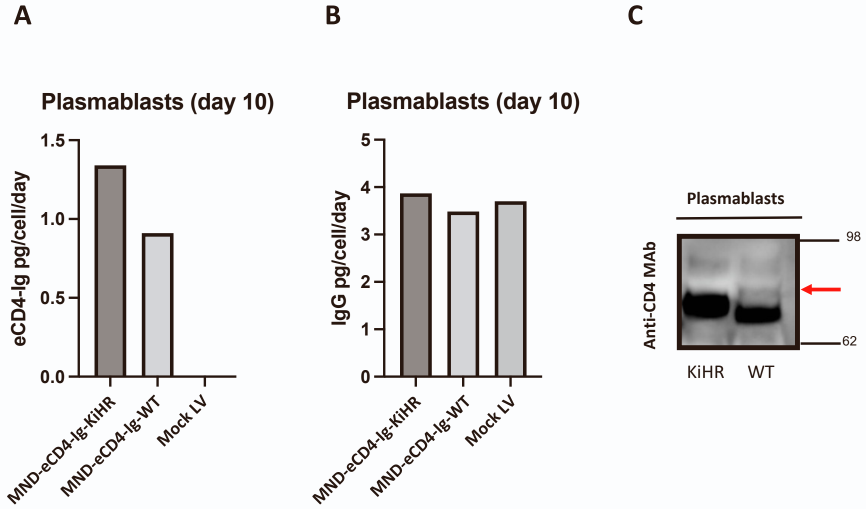

Figure S4. Production of eCD4-Ig-WT and eCD4-Ig-KiHR and IgG in LV transduced plasmablasts. [A] Measurements of eCD4-Ig and eCD4-Ig-KiHR and **[B]** IgG expression from plasmablast supernatants derived from primary B cells transduced with MV M-eGFP-eCD4-Ig-WT or MV M-eGFP-eCD4-Ig-KiHR LVs as described in Materials and Methods and **Figure 4**. IgG expression was measured with using an anti-IgG ELISA immunoassay and eCD4-Ig expression was quantified using an anti-gp140 ELISA assay. eCD4-Ig and IgG expression were normalized to the total number of live cells per day. **[C]** Detection of eCD4-Ig with the use of an anti-CD4 Mab non-reducing immunoblot. Red arrow identifies eCD4-Ig-WT and endogenous Ig Fc heterodimer association. B cells from PBMC of Donor 1.

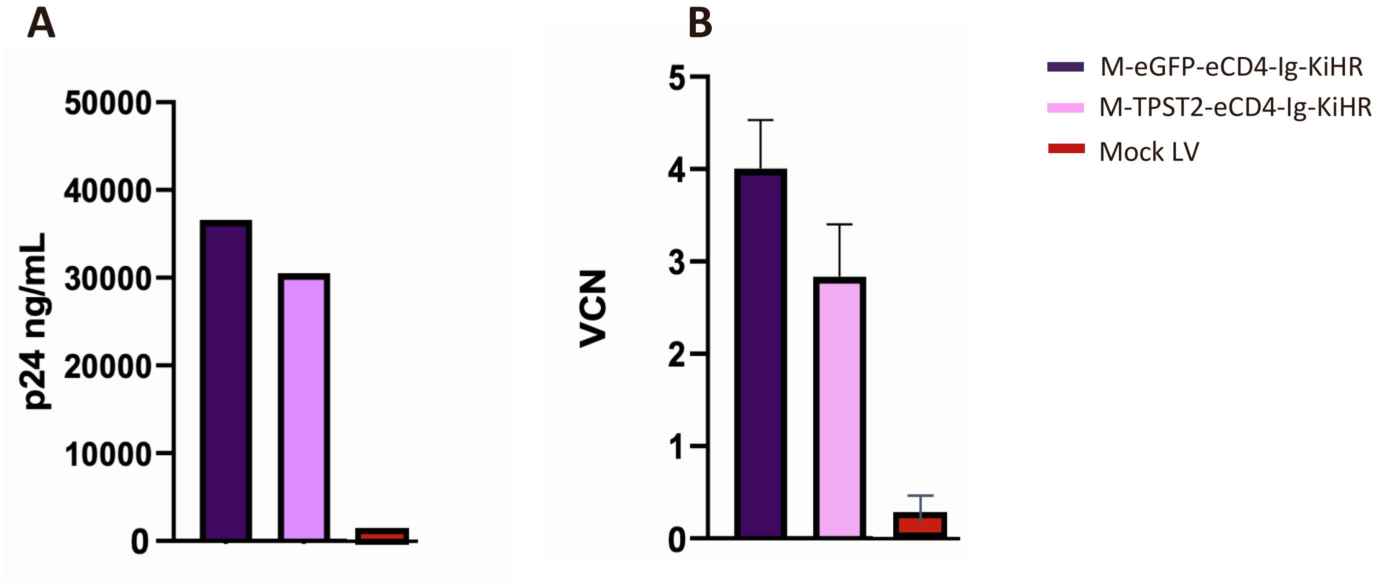

Figure S5. Characterization of MV M-eGFP-eCD4-Ig-KiHR and M-TPST2-eCD4-Ig-KiHR LV titers and B cell VCN after transduction. [A] ELISA p24 was performed on sucrose purified and concentrated MV M-eGFP-eCD4-Ig-KiHR and M-TPST2-eCD4-Ig-KiHR LV particles to measure physical titres. **[B]** MV-LVs were normalised for p24 content and used to transduce primary B cells (See **Figures 4, 5** for information on B donor and transduction protocols). B cell gDNA was extracted on the thirteenth day of the culture and samples were assessed for integrated provirus with digital droplet PCR (see Materials and Methods). Error bars show S.D. Wherever error bars are missing, it is due to lack of variability amongst duplicates.
