# Supplementary material for: A lentiviral vector B cell gene therapy platform for the delivery of the anti-HIV-1 eCD4-Ig-knob-in-hole-reversed immunoadhesin
Source: Mol Ther Methods Clin Dev. 2023 Feb 11;28:366–84. doi: 10.1016/j.omtm.2023.02.004 (PMC9984920; doi:10.1016/j.omtm.2023.02.004)
Supplement: Document S2. Article plus supplemental information [file mmc2.pdf]

Molecular Therapy
Methods & Clinical Development
Original Article

A lentiviral vector B cell gene therapy platform for the delivery of the anti-HIV-1 eCD4-Ig-knob-in-hole-reversed immunoadhesin

Eirini Vamva,[1,10] Stosh Ozog,[2,3,10] Daniel P. Leaman,[2] Rene Yu-Hong Cheng,[1] Nicholas J. Irons,[6] Andee Ott,[1] Claire Stoffers,[1] Iram Khan,[1] Geraldine K.E. Goebrecht,[2] Matthew R. Gardner,[7] Michael Farzan,[7] David J. Rawlings,[1,3,4,11] Michael B. Zwick,[2,11] Richard G. James,[1,3,5,11] and Bruce E. Torbett[1,2,3,8,9]

[1]Center for Immunity and Immunotherapies, Seattle Children's Research Institute, Seattle, WA, USA; [2]Department of Immunology and Microbiology, The Scripps Research Institute, La Jolla, CA, USA; [3]Department of Pediatrics, University of Washington School of Medicine, Seattle, WA, USA; [4]Department of Immunology, University of Washington School of Medicine, Seattle, WA, USA; [5]Department of Pharmacology, University of Washington School of Medicine, Seattle, WA, USA; [6]Department of Statistics, University of Washington, Seattle, WA, USA; [7]Department of Infectious Diseases, The Scripps Research Institute, Jupiter, FL, USA; [8]Department of Laboratory Medicine and Pathology, University of Washington, Seattle, WA, USA; [9]Institute for Stem Cell and Regenerative Medicine, Seattle, WA, USA

Barriers to effective gene therapy for many diseases include the number of modified target cells required to achieve therapeutic outcomes and host immune responses to expressed therapeutic proteins. As long-lived cells specialized for protein secretion, antibody-secreting B cells are an attractive target for foreign protein expression in blood and tissue. To neutralize HIV-1, we developed a lentiviral vector (LV) gene therapy platform for delivery of the anti-HIV-1 immunoadhesin, eCD4-Ig, to B cells. The EμB29 enhancer/promoter in the LV limited gene expression in non-B cell lineages. By engineering a knob-in-hole-reversed (KiHR) modification in the CH3-Fc eCD4-Ig domain, we reduced interactions between eCD4-Ig and endogenous B cell immunoglobulin G proteins, which improved HIV-1 neutralization potency. Unlike previous approaches in non-lymphoid cells, eCD4-Ig-KiHR produced in B cells promoted HIV-1 neutralizing protection without requiring exogenous TPST2, a tyrosine sulfation enzyme required for eCD4-Ig-KiHR function. This finding indicated that B cell machinery is well suited to produce therapeutic proteins. Lastly, to overcome the inefficient transduction efficiency associated with VSV-G LV delivery to primary B cells, an optimized measles pseudotyped LV packaging methodology achieved up to 75% transduction efficiency. Overall, our findings support the utility of B cell gene therapy platforms for therapeutic protein delivery.

INTRODUCTION

Human immunodeficiency virus (HIV-1) infection remains a global public health challenge, with approximately 38 million people worldwide living with HIV-1/AIDS.[1] While anti-retroviral therapy has had life-saving impacts on controlling existing infections, new strategies are still needed to provide for a functional HIV-1 cure. To eliminate the need for indefinite anti-retroviral treatment, many groups have attempted to create HIV-1-resistant $CD4^+$ T cells through gene delivery to either T cells or $CD34^+$ hematopoietic stem and progenitor cells (HSPCs).[2–7] The sizable number of HIV-1 susceptible target cells that must be genetically modified to provide protection and the possibility of limited expansion of genetically modified HIV-1 resistant cells in patients could constitute barriers to clinical anti-HIV-1 gene therapy implementation.[8,9] Therefore, alternative strategies focusing on engineering cells to secrete potent anti-viral therapeutic proteins may have therapeutic value.

Antibody gene transfer immunoprophylaxis, which involves viral delivery of genes that encode broadly neutralizing antibodies (bNAbs),[10–15] has been shown to be a promising approach. Furthermore, reports have described bNAb gene delivery to stem and progenitor cells in humanized mice.[16] Directed delivery of bNAb genes has been shown to be a protective strategy for simian-HIV (SHIV) challenges,[10,17,18] although it did not reproducibly decrease HIV-1 load despite measurable antibodies in people from injections of 10^{14} adeno-associated viral (AAV) genomes.[19] However, a recent report of a phase I AAV8 muscle delivery of VRC07 produced slightly more than 1 μg/mL in sera in volunteers at the peak, without a change in viremia, and with levels decreasing over the 3-year study period.[11] Given that many bNAbs used in monotherapy require high circulating concentrations to prevent viremia, >5 μg/mL, higher-affinity bNAbs are likely needed to prevent viremia.[12,20–23] Directed B cell immunoglobulin gene engineering to introduce novel anti-HIV paratopes has the distinct advantage that class switching and affinity maturation can occur, thereby potentially improving anti-HIV-1

Received 1 March 2022; accepted 8 February 2023;
https://doi.org/10.1016/j.omtm.2023.02.004.

[10]These authors contributed equally

[11]These authors contributed equally

Correspondence: Bruce E. Torbett, Center for Immunity and Immunotherapies, Seattle Children's Research Institute, Seattle, WA, USA.
E-mail: betorbet@uw.edu

 © 2023 The Author(s).
This is an open access article under the CC BY license (http://creativecommons.org/licenses/by/4.0/).

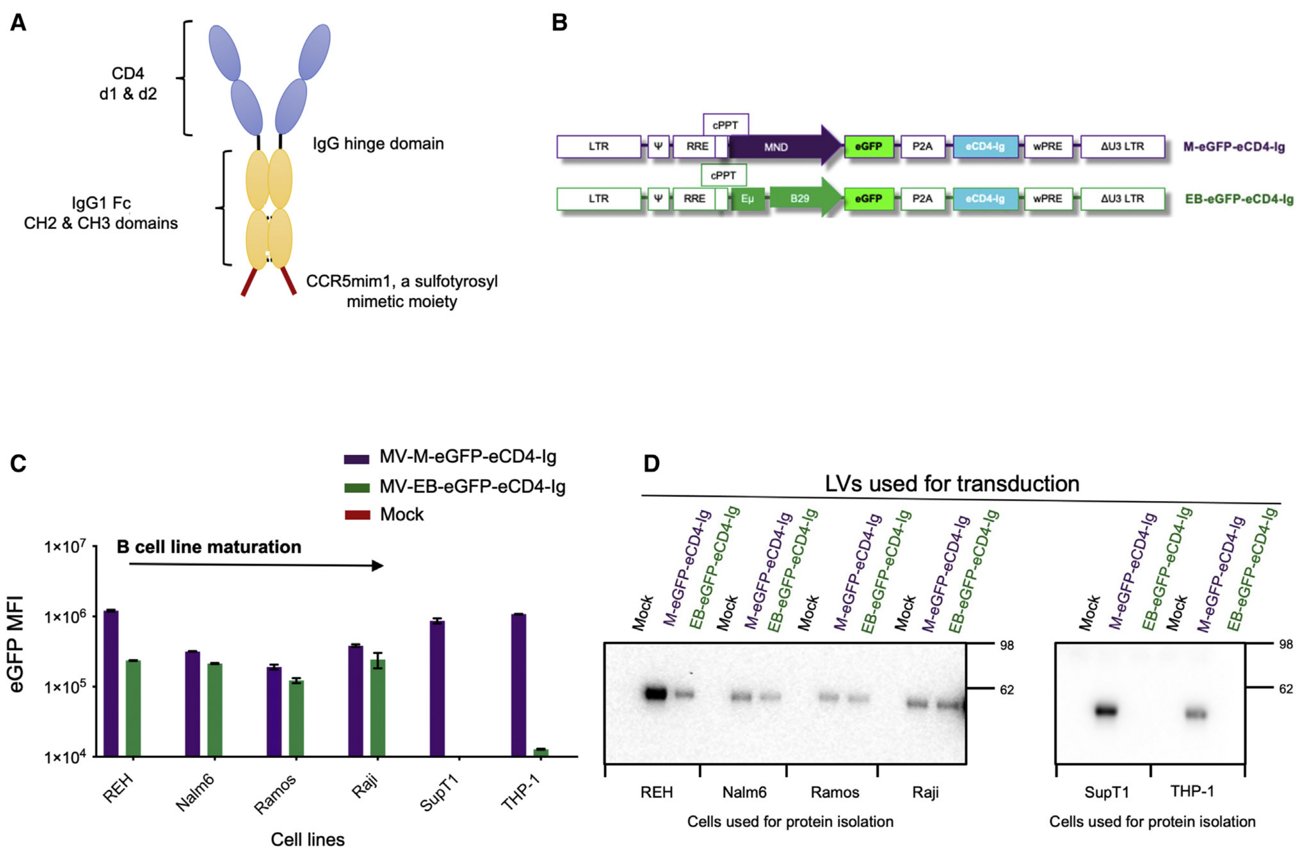

Figure 1. The B cell EμB29 promoter confers B cell lineage eCD4-Ig expression

(A) Schematic of the eCD4-Ig protein structure. (B) Schematics of lentiviral vectors (LVs) with constitutive MND (M-eGFP-eCD4-Ig) promoter or EμB29 B cell lineage (EB-eGFP-eCD4-Ig) enhancer/promoter regulating eGFP and eCD4-Ig expression. The schematic defines the *cis*-acting elements and open reading frames (ORFs) in the following order: the 5′ long terminal repeats (LTRs), the packaging *psi* element (Ψ), the Rev response element (RRE), the central polypurine tract (cPPT), each respective promoter, the eGFP and eCD4-Ig ORFs separated by a P2A self-cleaving peptide, the woodchuck hepatitis virus post-transcriptional regulatory element (WPRE), and the 3′ LTR containing a deletion in the U3 region (ΔU3 LTR). (C) Mean fluorescence intensity (MFI) of eGFP expression from human B, T (SupT1 $CD4^+$ T), and myeloid (THP-1) cell lines transduced with M-eGFP-eCD4-Ig (purple) or EB-eGFP-eCD4-Ig (green). Flow cytometry was performed 7 days post LV transduction. B cell lines assessed were of different maturation stages. (D) Assessment of eCD4-Ig production in culture supernatants from B cell, T cell, and myeloid cell lines 7 days post mock, M-eGFP-eCD4-Ig, or EB-eGFP-eCD4-Ig LV transduction. eCD4-Ig was detected using SDS-PAGE and anti-IgG western blot analysis. Bar graphs are mean ± SD of triplicates of representative experiments.

breadth and neutralization potency.[24–28] However, an ongoing concern in vaccine development and the use of a single bNAb for therapy remains the potential for HIV-1 resistance.[24,25] To limit HIV-1 escape, there are ongoing discussions to deliver bNAb cocktails with bNAbs of differing specificity with the intent to limit resistance development.[24,25]

Given these concerns, strides have been made to develop therapeutic inhibitor proteins with synthetic antibody-based architectures and increased affinity and breadth of neutralizing activity against HIV-1. A novel protein for targeting HIV-1 has been the development of immunoadhesins,[29] which are engineered immunoglobulin (Ig)-like structures that are less prone to engender HIV-1 resistance development.[29–31] One such protein is the eCD4-Ig immunoadhesin, a synthetic HIV-1 entry inhibitor which consists of domains 1 and 2 of CD4, the CH2 and CH3 domains of IgG1 Fc, and a short tyrosine-sulfated co-receptor mimetic peptide[31,32] (Figure 1A). eCD4-Ig differs from bNAbs given it binds two sites on the HIV-1 envelope which coincide with the CD4- and the HIV-co-receptor sites, resulting in an anti-HIV-1 immunoadhesin with exceptional breadth and potency of neutralization against a vast diversity of HIV-1 isolates.[32,33] Lower rates of HIV-1 resistance to eCD4-Ig, as compared with bNAbs, was reported.[33] Furthermore, HIV-1 mutants with partial resistance to eCD4-Ig were less fit.[33] Lastly, eCD4-Ig AAV-mediated direct delivery to non-human primate myocytes has been previously shown to control SHIV challenge.[32,34]

Primary plasma B cells can persist for a life span, which may provide a cell therapy advantage given that administration of B cells engineered to produce *de novo* proteins could provide long-term therapeutic benefit for a range of diseases that currently require multiple injections.[35] In addition, we have found that engineered plasma B cells

migrate to secondary lymphoid organs in the body and can produce localized proteins *in situ* for longer than 1 year.[36] Upon activation, B cells can differentiate into long-lived plasma cells that secrete large quantities of antibodies, making them an excellent candidate for gene-therapy-mediated immunotherapy approaches.[37–39] B cells also provide a promising approach for active immunotherapy against pathogens, such as HIV-1, given their ability to secrete high quantities of antibody and to migrate into most tissues.[40] Despite the great potential of B cells for gene therapy applications, their use has been limited by technical challenges including inconsistent/inefficient gene delivery and packaging capacity of gene-editing reagents.[35,41,42] Lentiviral vectors (LVs) appear to offer a safe integration profile combined with stable transgene expression and have a large packaging capacity.[43] However, VSV-G pseudotyped LVs are inefficient at transducing primary human B cells.[41] Developed alternative LV pseudotypes can achieve higher B cell transduction rates (up to ~50%) but have lower viral titers, which make inclusion of larger transgenes and large-scale production challenging.[44–47]

Here, we report on the development and evaluation of an LV gene therapy platform for primary human B cell delivery of the eCD4-Ig immunoadhesin.[32] Our optimized measles pseudotyped LV transduction protocol achieved *in vitro* delivery of eCD4-Ig transgene delivery of ~75% of B cells. Upon LV cellular integration, we demonstrate expression of eCD4-Ig in B cells using the B cell EμB29 enhancer/promoter.[48] Next, we developed a knob-in-hole-reversed (KiHR) design to prevent or limit heterodimer formation between the Fc domains in eCD4-Ig and endogenous B cell IgGs. The eCD4-Ig-KiHR promoted homodimer formation in the presence of cytoplasmic B cell IgGs and increased anti-HIV-1 potency. Lastly, we show that unlike AAV delivery to non-lymphoid tissues,[32] co-expression of the tyrosylprotein sulfotransferase 2 (TPST2) a tyrosine sulfation enzyme,[49] necessary for eCD4-Ig HIV-1 neutralization function, was not needed for efficient HIV-1 neutralization when eCD4-Ig was produced by primary B cells. These improvements in B cell gene delivery, expression, and eCD4-Ig-KiHR protein design provide a potential B cell gene therapy platform for eCD4-Ig-KiHR production. Moreover, we propose that this B cell delivery platform could be adapted for regulated protein production for a range of therapeutic applications to combat human diseases.

RESULTS

Establishment of a B cell lineage lentiviral vector for eCD4-Ig expression

A subset of antibody-secreting B cells are composed of long-lived, protein-producing cells, with the ability to home to and reside in the bone marrow or in localized sites of infection. We hypothesized that these cellular attributes might be ideal for stable production of eCD4-Ig (Figure 1A). Therefore, in this study we sought to develop an efficient LV delivery system for eCD4-Ig gene to B cells. We first tested alternative promoters for use in LV eCD4-Ig expression: (1) a constitutive MND promoter (M), which has been shown to mediate high-level expression in all hematopoietic lineages, including B cells;[50,51] and (2) the EμB29 enhancer/promoter (EB), previously shown to mediate high levels of transgene expression in B cell lines, with limited expression in other lymphoid and myeloid cells.[48] The bicistronic LVs developed contained eCD4-Ig and eGFP, a reporter molecule for monitoring gene expression[52] separated by the P2A peptide that enables self-cleavage by ribosomal skipping,[53] thus yielding two separate proteins (Figure 1B).

To compare the LV M promoter and EB promoter/enhancer for their expression of eCD4-Ig and eGFP, B cell lines of various stages of differentiation, a $CD4^+$ T cell line (SupT1), and a myeloid cell line (THP-1) were transduced with VSV-G LVs, matched for an integrated viral copy number (VCN) of approximately 1 (Figure 1C). Transduction with EB-eGFP-eCD4-Ig LVs showed low mean fluorescence intensity (MFI) eGFP expression in transduced THP-1 cells and robust expression in all B cell lines. In contrast, all cell types transduced with M-eGFP-eCD4-Ig LVs exhibited robust eGFP MFI. We next quantified eCD4-Ig production from stably LV transduced cell supernatants by anti-IgG western blot analysis (Figure 1D). In contrast to the B cell lines, eCD4-Ig was not observed in EB-eGFP-eC4-Ig transduced SupT1 or THP-1 cells. Consistent with the eGFP findings and our earlier report,[48] these findings suggest that the EμB29 enhancer/promoter optimally expresses in B cells.

To determine whether enhancing LV transduction levels influenced EμB29 enhancer/promoter activity and promoted eCD4-Ig production in non-B cell lineages, we utilized small molecules previously shown to facilitate LV transduction in $CD34^+$ HSPCs, the cyclic polyphenol compound caraphenol A (cara)[54] and prostaglandin E2 (PGE-2).[55] UCB-derived $CD34^+$ HSPCs were transduced with M-eGFP-eCD4-Ig and EB-eGFP-eCD4-Ig LV, either in DMSO as a vehicle control, cara, PGE-2, or both small molecules (Figure S1A). The expression of eGFP observed in M-eGFP-eCD4-Ig LV stably transduced cells at day 7 (Figure S1A) was similar to eGFP MFI expression intensity as observed in the REH B cell line, $\sim 10^6$ MFI (Figure 1C). In contrast, eGFP MFI expression was $\sim 2 \log_{10}$ lower in EB-eGFP-eCD4-Ig LV stably transduced $CD34^+$ cells when compared with M-eGFP-eCD4-Ig LV transduced $CD34^+$ cells, despite a progressive increase in VCN due to transduction enhancers (Figure S1B). The findings of low-level eGFP MFI expression in all $CD34^+$ cells (Figure S1A) were similar in eGFP MFI expression intensity to THP1 myeloid cells (Figure 1C). The low-level eGFP MFI expression may be the result of the reported presence of $CD19^+$ pre-B lymphocytes within the cord blood $CD34^+$ population.[56] In addition, the transcription factor PU.1, found in B cells and myeloid cells,[57] activates B29.[58] Regardless of the low-level eGFP MFI expression observed in $CD34^+$ cells stably transduced with EB-eGFP-eCD4-Ig LVs, the resulting EμB29 enhancer/promoter activity was not sufficient to produce detectable eCD4-Ig (Figure S1C). In contrast, $CD34^+$ cells stably transduced with LV-M-eGFP-eCD4-Ig LV produced detectable eCD4-Ig, confirming that $CD34^+$ cells have the capacity to produce eCD4-Ig (Figure S1C). The B cell, T cell, and myeloid cell lines and $CD34^+$ cell evaluations confirmed that the EμB29 enhancer/promoter activity was not sufficient in non-B cells to produce secreted, detectable eCD4-Ig. Our findings support

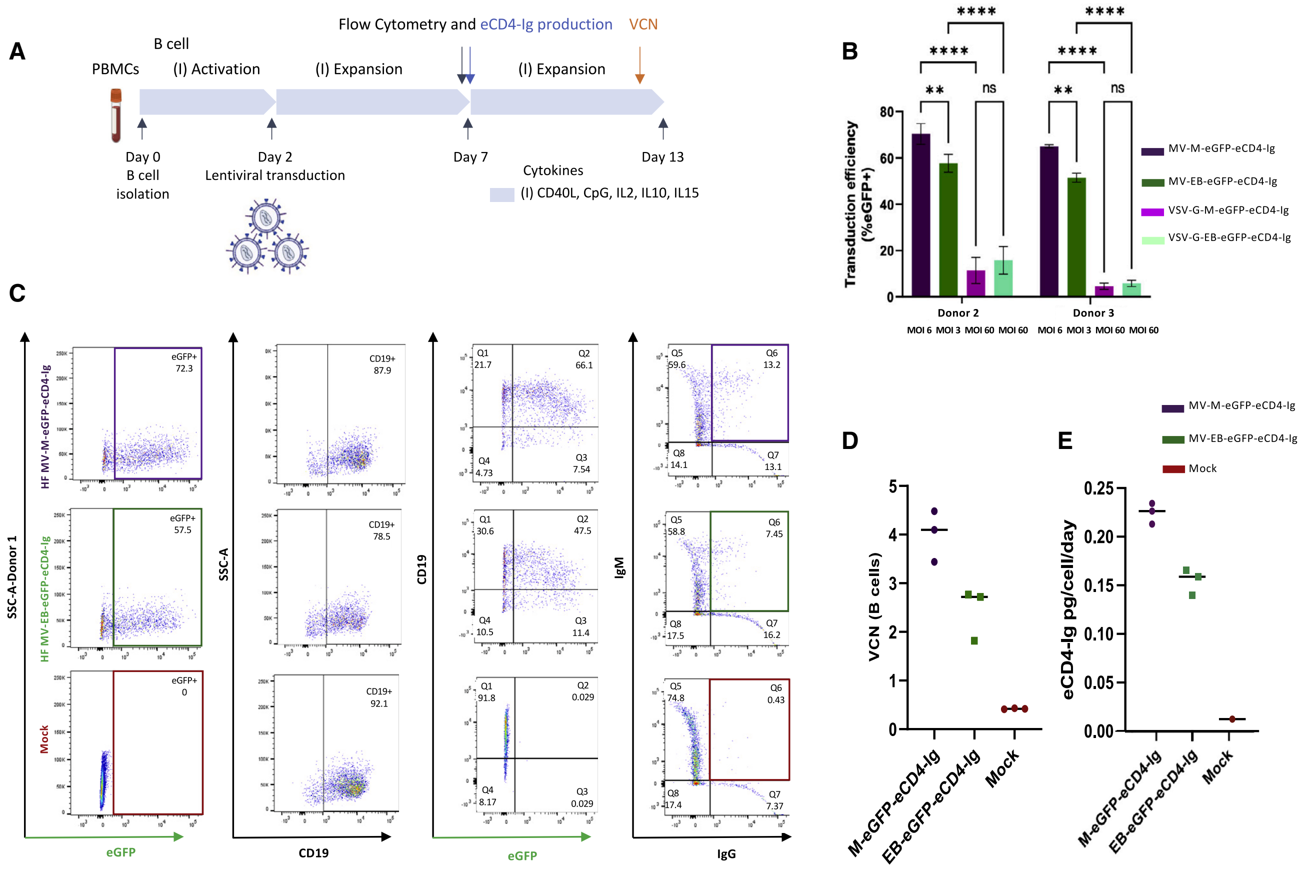

Figure 2. Optimization of measles pseudotyped lentiviral vector transduction of primary human B cells

(A) Schematic of the workflow and timeline of the B cell studies performed. Primary B cells were isolated from PBMCs of donors 2 and 3. See materials and methods for culture conditions, assays, flow cytometry, and vector copy number per cell (VCN) information. (B) Comparison of B cell transduction efficiencies of MV-M-eGFP-eCD4-Ig, MV-EB-eGFP-eCD4-Ig, VSV-G-M-eGFP-eCD4-Ig, and VSV-G-EB-eGFP-eCD4-Ig LVs. LV transduction efficiency was assessed by flow cytometry for eGFP 5 days post transduction (n = 3, mean and SD are shown; ns, not significant; $^{**}p < 0.01$, $^{****}p < 0.0001$). Donors 2 and 3 are shown. (C) Representative flow-cytometric analysis of B cells transduced with MV-M-eGFP-eCD4-Ig, MV-EB-eGFP-eCD4-Ig, and mock (no LV). B cells were assessed by flow cytometry for eGFP, CD19, IgM, and IgG expression 5 days post LV transduction. Donor 1 is shown. (D) Genomic DNA was extracted 11 days post LV transduction from both B cell donor cells to assess the VCN. (E) Supernatants from LV transduced primary B cells were harvested 5 days post LV transduction (expansion period day 7) and assessed for eCD4-Ig production. eCD4-Ig production was normalized to the total number of live cells per day post transduction. Data presented are mean ± SD of triplicates, with the ranges shown.

the use of the EμB29 enhancer/promoter for B cell expression of eCD4-Ig in primary B cells.

Evaluation of measles pseudotyped LV for efficient human B cell transduction

To evaluate the production of eCD4-Ig following LV transduction in primary human B cells, we adapted culture methods previously optimized for human B cell editing[59] to our recently reported protocol to optimize LVs pseudotyped with the measles hemagglutinin and fusion (H/F) proteins for B cell transduction.[60] Primary B cells were isolated from peripheral blood mononuclear cells (PBMCs) using negative selection, activated with a custom cytokine cocktail, and transduced with volume-matched LV *ex vivo* (Figure 2A). We initially compared the rates of B cell transduction of eCD4-Ig and *cis*-linked GFP via the commonly used VSV-G LV or measles virus pseudotyped LV (MV) (Figure 2B). Although the concentrated titers for the MV LVs were lower than VSV-G LVs using the Nalm6 B cell line for titering (Figure S2A), we found that MV LVs exhibit significantly better transduction of primary human B cells despite being delivered at ∼10-fold lower multiplicities of infection (MOIs) than VSV-G LVs (Figure S2B). We used volume M LV comparisons for transduction efficacy given the occasional observation of primary B cell toxicity. These findings support the use of MV pseudotyped LVs for primary B cell gene delivery. Consequently, all subsequent B cell gene delivery studies in the report employed the use of the MV LV system.

Next, we evaluated MV-M-eGFP-eCD4-Ig (MOI = 6), MV-EB-eGFP-eCD4-Ig (MOI = 3) VSV-G-M-eGFP-eCD4-Ig (MOI = 60), and VSV-G-EB-eGFP-eCD4-Ig (MOI = 60) primary B cell transduction using established B cell culture conditions (Figure 2A) and

assessed eGFP frequency in B cell samples from two additional human donors 5 days post transduction (Figure 2B). Again, MV LV was superior to VSV-G LV for transducing activated B cells, MV LVs providing 4- to 33-fold greater transduction efficacy than VSV-G LV (Figure 2B) depending on the B cell donor sample and LV MOI. We then followed up with flow cytometry and B cell subset discrimination with CD19, IgG, and IgM 5 days post transduction (Figure 2C). LV transduction and eCD4-Ig expression did not appear to alter the proportion of $CD19^+$ B cells relative to mock controls. Notably, transduction with either MV-M-eGFP-eCD4-Ig or MV-EB-eGFP-eCD4-Ig led to generation of a dual-positive IgG^+IgM^+ population based upon intracellular staining, consistent with eCD4-Ig expression in IgM^+ B cells. The average transduction frequency and VCN for MV-LV-M-eGFP-eCD4-Ig and for MV-LV-EB-eGFP-eCD4-Ig were 71.4% and 4.1, and 50.7% and 2.7, respectively (Figures 2B and 2D). Secreted eCD4-Ig protein was evident in the culture supernatant and correlated with VCN levels, with MV-LV-M-eGFP-eCD4-Ig producing 900 ng/mL/~500,000 cells and MV-LV-EB-eGFP-eCD4-Ig producing 500 ng/mL/~500,000 cells (Figure 2E). Together, these findings demonstrate that MV LVs allowed for efficient primary B cell transduction (Figure 2B) and integration, as judged by VCN (Figure 2D) within a clinically acceptable range[61] and resulted in robust eCD4-Ig production (Figure 2E). These findings support the use of this optimized MV LV system for delivery of eCD4-Ig to primary human B cells.

Development of a knob-in-hole-reversed eCD4-Ig variant for improved B cell expression

The ability of eCD4-Ig to dimerize, persist in serum, and interact with cellular Fc receptors to initiate antibody-dependent cell cytotoxicity (ADCC) depends on its IgG Fc domain.[62] Endogenous IgG is one of the most highly expressed proteins in plasma B cells. Given the shared CH2 and CH3 in eCD4-Ig and endogenous IgGs, there is a potential to heterodimerize. Heterodimerization could reduce eCD4-Ig production and secretion and/or its HIV-1 neutralization function efficiency. To test for heterodimerization, plasmids containing IgG heavy- and light-chain genes for the anti-HIV bNAb VRCO1[22] and eCD4-Ig were co-transfected into HEK293T cells, and supernatants were queried using anti-IgG western blotting (Figure 3A). In non-reducing conditions, we observed the eCD4-Ig homodimer (~80 kDa), the VRCO1 homodimer (~150 kDa), and a third band (~125 kDa; red arrow) that was consistent with a heterodimer composed of VRCO1 and eCD4-Ig. An illustration is provided below the gel blots representing the observed outcome of cellular co-expression of VRCO1 IgG and eCD4-Ig, as well as individual expression of VRC01 IgG and eCD4-Ig, as revealed on the gel (Figure 3A).

We reasoned that modification of the Fc Ig domain in eCD4-Ig could be utilized to limit heterodimerization with endogenous B cell IgGs. Structural manipulation of the Fc dimeric interface has long been used to promote heterodimerization in the production of bispecific antibodies through the introduction of complementary amino acids that sterically clash when homodimerized.[63] The knob-in-hole (KiH) concept is an established method used to pair large side-chained amino acids on one chain with smaller side chains on the intended partner dimer.[64] Through a combination of structural and sequence-based computational approaches, Moore et al.[65] had previously identified T394F and F405A as lead variants that can dramatically improve bispecific antibody heterodimerization (to alter Fc selection for desired Fcs), while decreasing homodimerization, when individually introduced into each IgG CH3 domain. Relying on the symmetry of the Fc CH3 domain in eCD4-Ig dimers (Figure 1A), our structural-based engineering strategy was to reverse this previously reported KiH approach (i.e., KiHR)[66] by introducing both the T394F and F405A mutations together into the CH3 domain of eCD4-Ig (Figure 3B). Our expectation was that the bulky T394F side chain in the CH3 domain of eCD4-Ig would clash with the endogenous IgG CH3 domain F405 (bulky side chain), potentially reducing pairing (Figure 3B). Yet the CH3 eCD4-Ig domain mutation would favor eCD4-Ig homodimerization, given that the F405A mutation would not sterically clash with eCD4-Ig CH3 domains, thereby maintaining preferred eCD4-Ig homodimerization (Figure 3B).

To test whether our predicted eCD4-Ig Fc domain KiHR modification disrupts IgG heterodimerization with eCD4-Ig, HEK293T cells were co-transfected with plasmids containing the eCD4-Ig-KiHR (T394F and F405A mutations) LVs and VRCO1 IgG for evaluation, as well as supernatants from plasmid control co-transfections of eCD4-Ig-WT (wild type) and VRCO1 IgG. Collected supernatants were subsequently analyzed by anti-IgG western blot under non-reducing conditions. In agreement with our previous findings (Figure 3A) an intermediate band suggestive of heterodimerization was observed again when using WT eCD4-Ig and VRCO1 IgG gel lanes (Figure 3C). In contrast, no intermediate band was detected in the gel lane when using the eCD4-Ig-KiHR variant with VRCO1 IgG (Figure 3C) lane. An illustration is provided below the gel blots representing the observed outcome of cellular co-expression of VRCO1 IgG and eCD4-Ig-KiHR, as well as individual cellular expression of VRC01 IgG and eCD4-Ig and cellular co-expression of VRCO1 IgG and eCD4-Ig, as revealed on the gel (see Figure 3A for comparisons). These findings suggest that the CH3 engineering of T394F and F405A mutations indeed promoted disfavored heterodimerization between eCD4-Ig and VRC01 and favored eCD4-Ig-KiHR homodimerization. Thus, our combined findings support the use of the eCD4-Ig-KiHR to prevent potential heterodimerization between eCD4-Ig and endogenous IgG in B cells.

LVs containing the KiHR CH3 domains LV-MND-eGFP-eCD4-Ig-KiHR and LV-EB-eGFP-eCD4-Ig-KiHR LV WT (Figure S3) were developed for evaluation. MV-LV-MND-eGFP-eCD4-Ig-KiHR and MV-LV-MND-eGFP-eCD4-Ig-KiHR were then used for transduction of Nalm6 B cell line (lacks IgG), primary activated B cells (medium levels of IgG production), and primary B cells differentiated to plasmablasts (high levels of IgG production) (Figures 4A and 4B). Anti-IgG and anti-CD4 western blotting of equal volumes of culture supernatants were employed to evaluate heterodimerization of eCD4-Ig and endogenous IgG. Under non-reducing conditions, the anti-IgG blots were predicted to show endogenous IgG heavy-chain

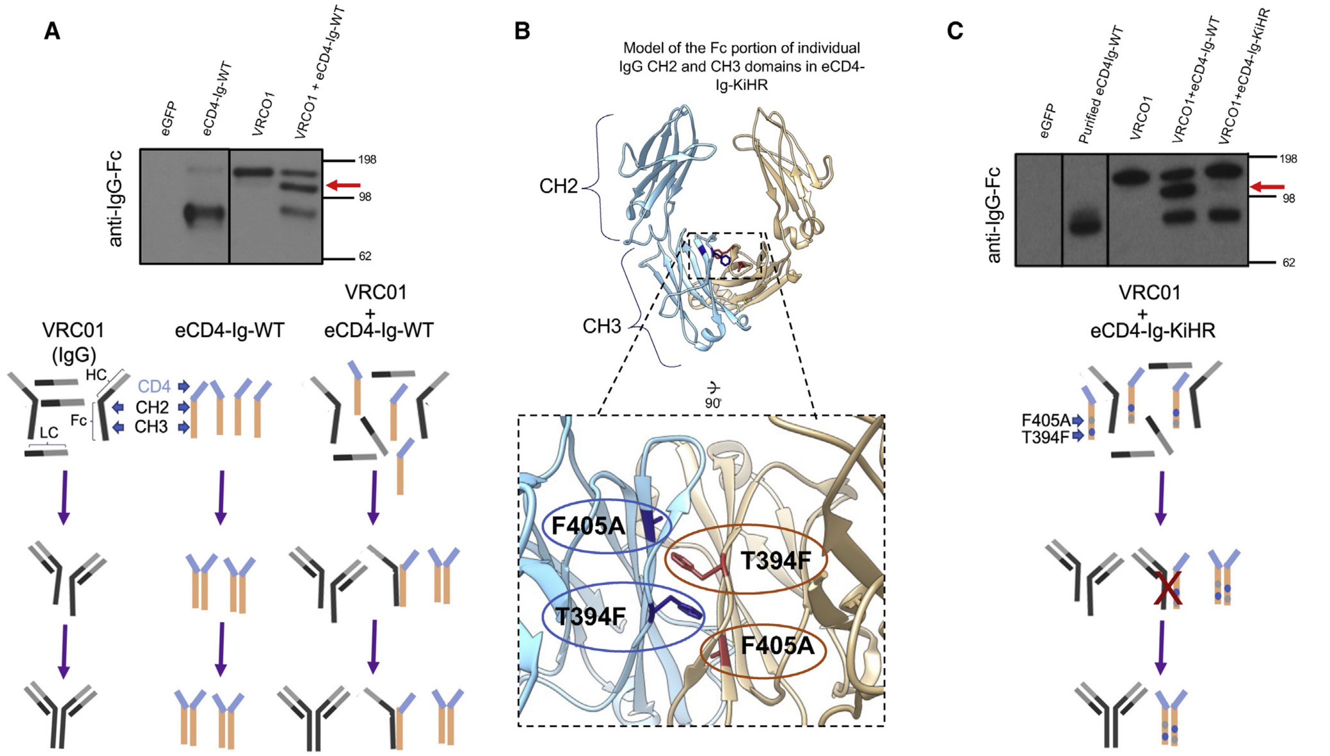

Figure 3. Knob-in-hole-reversed eCD4-Ig variant diminishes cellular eCD4-Ig recombination with co-expressed anti-HIV VRCO1 IgG1 bNAb

(A) A non-reducing anti-IgG western blot from supernatants obtained from transiently transfected HEK293T cells with eCD4-Ig (WT), the anti-HIV bNAb VRCO1 IgG, or co-transfection of both plasmids. The red arrow identifies the VRCO1 and eCD4-Ig heterodimer band. A pictorial representation of the gel findings is shown: HC, IgG heavy chain; LC, IgG light chain; Fc domain showing CH2 and CH3 domain. Figures are not to relative scale. (B) A ribbon structural model showing the individual monomers (blue and tan) of the Fc portion of CH2 and CH3 domains of eCD4-Ig (see also Figure 1A), in which the CH3 dimerization interface was engineered to construct the eCD4-Ig knob-in-hole-reversed (KiHR). Shown are the KiHR mutations F405A and T394F in the CH3 dimerization interface (red and blue colored residues) which are predicted to sterically clash with CH3 domains of non-KiHR CH3 antibody domains. See materials and methods for additional information. (C) A non-reducing anti-IgG western blot analysis of HEK293T cell supernatants from cells transiently transfected with the plasmids eCD4-Ig or VRCO1, or co-transfected with the VRCO1 and eCD4-Ig-WT or VRCO1 and eCD4-Ig-KiHR plasmid combinations. The red arrow identifies the VRCO1 and eCD4-Ig-WT heterodimer band in the western blot lane. Note the presence of independent VRCO1 IgG and eCD4-Ig-KiHR bands in the co-expressed VRCO1 and eCD4-Ig-KiHR lanes and the lack of a heterodimer band. An illustration below the gel figure is a pictorial representation of the gel findings for eCD4-Ig-KiHR and VCR01 IgG lane.

homodimer at ∼100 kDa, the eCD4-Ig homodimer at ∼75 kDa, and a heterodimer, if produced, at ∼87 kDa. Notably, under non-reducing gel running conditions the WT and KiHR eCD4-Igs were detected as different sizes, suggesting potential structural and/or folding differences between the two proteins. Furthermore, in activated B cells and plasmablasts expressing eCD4-Ig-WT, we observed a shift in size in the endogenous IgG band (Figure 4B, orange and purple arrows), potentially suggestive of heterodimerization. The anti-CD4 blot further confirmed the size-structure differences between the WT and KiHR eCD4-Igs and revealed increased detection of KiHR eCD4-Ig, despite similar detection of the Fc. These observations suggest potential differences in the availability of the CD4 domains resulting from the KiHR mutations promoting structural differences and/or heterodimerization.

We next tested whether the observed differences in eCD4-Ig heterodimerization would affect HIV-1 neutralization efficacy. To investigate this possibility, HIV-1 neutralization assays[67] were performed to assess the ability of eCD4-Ig, or a bNAb, to decrease HIV-1 infection of a susceptible target cell. Notably, the sulfonation of the CCR5mim1 sequence in eCD4-Ig (Figure 1A) is critical for efficient eCD4-Ig HIV-1 neutralization activity.[32,68] The mammalian sulfation enzyme, protein-tyrosine sulfotransferase 2 (TPST2),[49] required for sulfonation of eCD4-Ig, has previously been reported to be expressed in B cells.[69] Therefore, we predicted that B cell TPST2 enzymatic activity would be sufficient to sulfonate eCD4-Ig. To test for eCD4-Ig and eCD4-Ig-KiHR HIV-1 neutralization activity, culture supernatants were collected on day 10 from plasmablasts differentiated from primary B cells transduced with LV-M-eGFP-eCD4-Ig-KiHR, LV-M-eGFP-eCD4-Ig-WT, LV-EB-eGFP-eCD4-Ig-KiHR, LV-EB-eGFP-eCD4-Ig-WT, or mock LV (Figures 4A and 4C). Culture supernatants were then tested against three different HIV-1 isolates. The HIV-1 isolates used for evaluation were NL4-3 (CXCR-4 tropic isolate, clade B, tier 1A, T cell line adapted strain), JRFL (CCR5 tropic

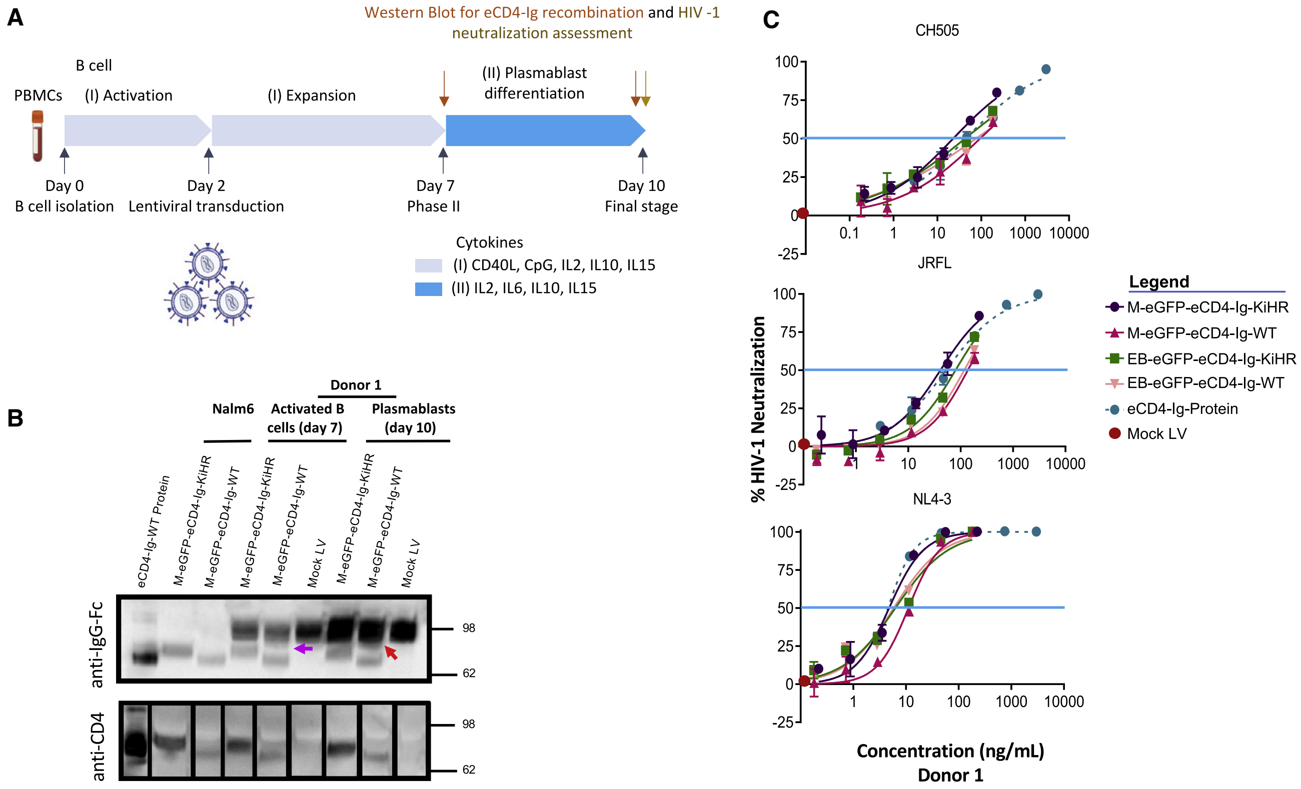

Figure 4. Assessment of eCD4-Ig and eCD4-Ig KiHR expression in primary B cells, and the capacity of B cell secreted eCD4-Ig and eCD4-Ig KiHR to neutralize HIV-1

(A) Schematic of the workflow of B cell studies performed. See materials and methods for culture condition information. B cells were from donor 1. (B) Non-reducing western blot analysis for detection of IgG and CD4 from supernatants of Nalm6 cells, activated B cells, and differentiated plasmablasts transduced with the LVs listed (see legend). Supernatants were harvested 3 days post transduction from Nalm6 cells, for expanded B cells on day 7 and plasmablasts on day10. Twenty microliters of supernatants from cell culture was loaded for analysis, recombinant eCD4-Ig was used as a positive control, and supernatants from mock LV transduced activated B cells and differentiated plasmablasts were used as negative controls. Red arrow identifies heterodimer association. (C) HIV-1 neutralizing capacity of day-10 B cell plasmablast supernatants from B cells transduced with listed LVs or no LV (mock). Cell supernatants were evaluated for HIV-1 neutralizing potency against the HIV-1 isolates NL4-3, JRFL, and CH505, as described in materials and methods. Purified recombinant eCD4-Ig was used as a positive control, and supernatant from mock LV transduced B cells was used as a negative control. Blue horizontal line marksIC_{50}, and graph points show range and average of duplicates. The lack of sample assay range is due to lack of variance between individual assays. IC_{50}s were calculated from six dilution points of each supernatant. Paired one-sided Welch's t test was used for analysis; IC_{50} HIV-1 neutralization assessment of n = 6 eCD4-Ig-WT and n = 6 eCD4-Ig-KiHR repeats, with three repeats for each LV promoter, $p < 0.022$.

isolate, clade B, tier 2), and CH505 (a primary-like CCR5 tropic isolate, clade C, tier 2 transmitted founder isolate).

An important first observation was that B cells stably expressing the eCD4-Ig and eCD4-Ig-KiHR genes produced protein that neutralized HIV-1 across all clades and co-receptor dependencies (Figure 4C). Based on eCD4-Ig and eCD4-Ig-KiHR HIV-1 neutralization functionality, TPST enzymatic activity was present in plasmablasts. Furthermore, the neutralization assays IC_{50} assessment (blue line in Figure 4C) of different combinations of LV promoters and HIV-1 isolates revealed that the KiHR variant mildly outperformed WT eCD4-Ig regardless of HIV strain or LV promoter ($p < 0.022$). Additionally, eCD4-Ig-KiHR cellular protein production (Figures S4A and S4C) was modestly improved for the eCD4-Ig-KiHR variant as compared with the WT eCD4-Ig, with no apparent effect on IgG production (Figure 4B). Overall, the combined findings indicate that the eCD4-Ig-KiHR protein decreased heterodimer formation and modestly increased protein production in B cells as well as producing more efficient HIV-1 neutralization activity. Based on the superior performance, all subsequent studies relied on the KiHR-engineered Fc variant.

Endogenous B cell TPST2 is sufficient for optimal eCD4-Ig-KiHR HIV-1 neutralization function

In previous AAV-delivered eCD4-Ig immunoprophylaxis studies in non-human primate myocytes, co-delivery of TPST2 was utilized to promote eCD4-Ig sulfation and efficient HIV-1 neutralization.[32] Therefore, although B cell production of eCD4-Ig-KiHR was robust (Figure S4A) and neutralized HIV-1 (Figure 4C), we next tested whether ectopic co-expression of TPST2 might further increase the

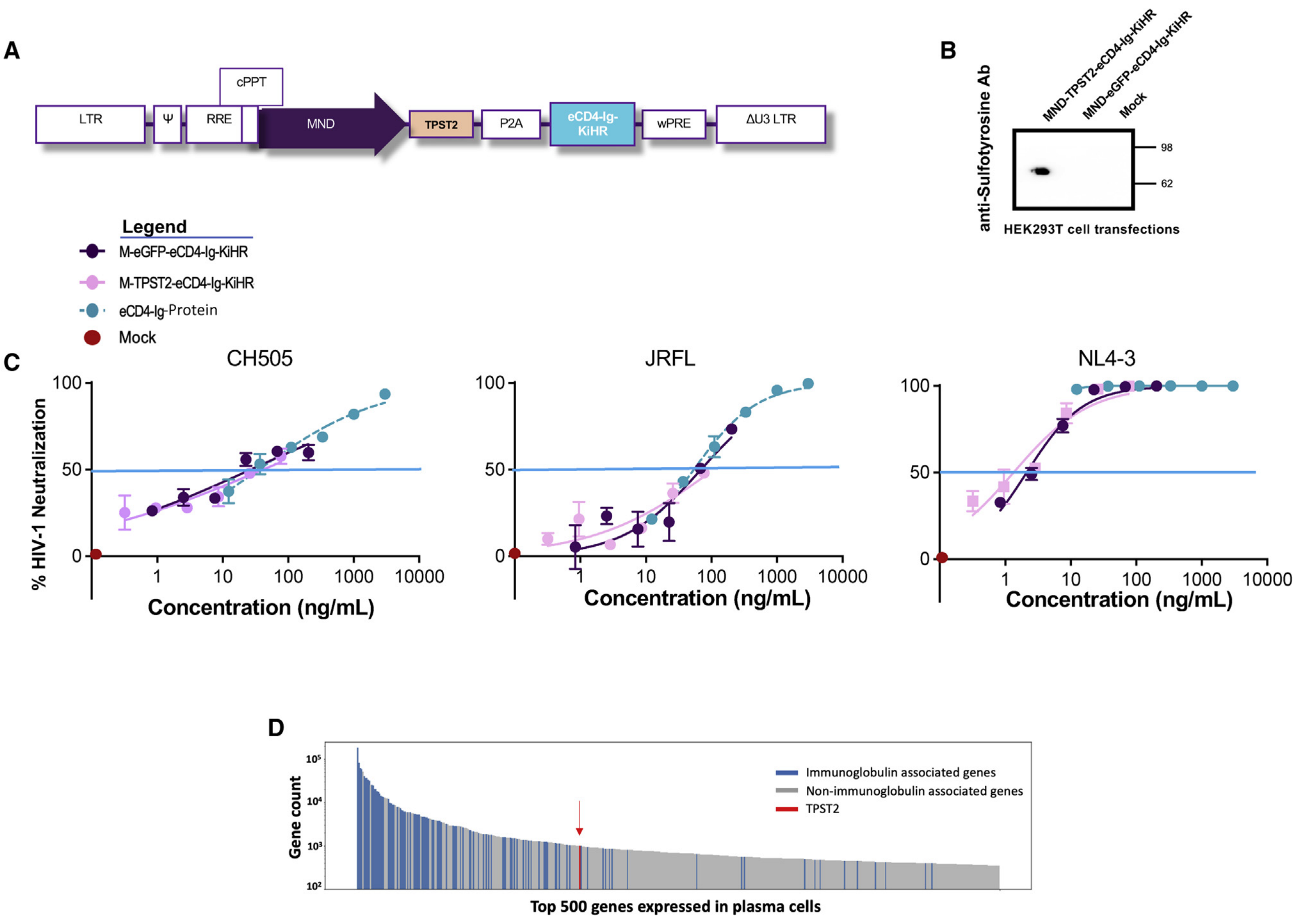

Figure 5. Ectopically expressed TPST2 in primary B cells does not improve eCD4-Ig-KiHR HIV-1 neutralization functionality

(A) TPST2-P2A-eCD4-Ig LV schematic with the constitutive (MND-M) promoter. The LV elements are as described in Figure 1B. (B) Representative anti-sulfotyrosine non-reducing western blot analysis from supernatants of HEK293T cells transfected with the pM-TPST2-eCD4-Ig-KiHR or pM-rGFP-eCD4-Ig-KiHR LV plasmids. Supernatants were harvested 4 days post transfection and evaluated to verify the presence of eCD4-Ig-KiHR using an anti-CD4 ELISA assay as described in materials and methods. Next, supernatants containing equal amounts of eCD4-Ig-KiHR (20 mg) were analyzed by SDS-PAGE and western blot analysis using anti-sulfotyrosine antibody to detect sulfonylation of eCD4-Ig-KiHR. (C) Stable LV transduction of primary B cells with M-TPST2-eCD4-Ig-KiHR LV did not enhance the HIV-1 neutralization functionality of eCD4-Ig-KiHR. B cell cultures were established as described in Figure 4A, and on day 10 culture supernatants were collected and evaluated for eCD4-Ig-KiHR amounts using anti-gp140 ELISA. HIV-1 neutralization assays were performed as described in Figure 4C and materials and methods, using equal amounts of eCD4-Ig-KiHR-containing supernatants to evaluate the effect of ectopically expressed TPST2 on eCD4-Ig-KiHR HIV-1 neutralization functionality. Purified eCD4-Ig was used as a positive control, and supernatant from mock untransduced B cells was used as a negative control. Blue horizontal line marks IC_{50}, and graph points show range and average of duplicates. Note overlapping IC_{50S} intersect (blue line)for all HIV-1 isolates and all are within the assay variance. IC_{50}s were calculated from six dilution points of each supernatant. (D) TPST2 is highly expressed in B cells. RNA sequencing was performed on $CD138^+$ cells enriched from human plasma cell cultures. Raw counts were averaged for the top 500 expressed genes (n = 4 donors), and the results are plotted. Red arrow identifies TPST2 gene location.

eCD4-Ig HIV-1 neutralization activity of LV transduced B supernatants. Thus, we wondered whether robust ectopic eCD4-Ig-KiHR protein expression in B cells overwhelmed TPST2 enzymatic activity. To address this question, TPST2 sequences[32] were introduced in a bicistronic context to generate M-TPST2-eCD4-Ig-KiHR LV (Figure 5A). This construct was first tested by plasmid transfection using M-TPST2-eCD4-Ig-KiHR and M-eGFP-eCD4-Ig-KiHR, as a control, in HEK293T cells, reported to have undetectable endogenous TPST activity.[70] Western blot analysis demonstrated sulfonation activity only in supernatants derived from M-TPST2-eCD4-Ig-KiHR transfected cells (Figure 5B), validating that M-TPST2-eCD4-Ig-KiHR produced enzymatically active TPST2.

To evaluate the effect of ectopically expressing TPST2 in B cells and the resulting effect on eCD4-Ig-KiHR HIV-1 neutralizing functionality, day-10 supernatants from plasmablasts transduced (as described in Figure 4A) with an equivalent MOI of MV-M-eGFP-eCD4-Ig-KiHR or MV-M-TPST2-eCD4-Ig-KiHR (Figure S5) were assessed in HIV-1 neutralization assays, as described in Figure 4C. We found that the IC_{50} values for neutralization of different HIV-1 isolates by eCD4-Ig-KiHR

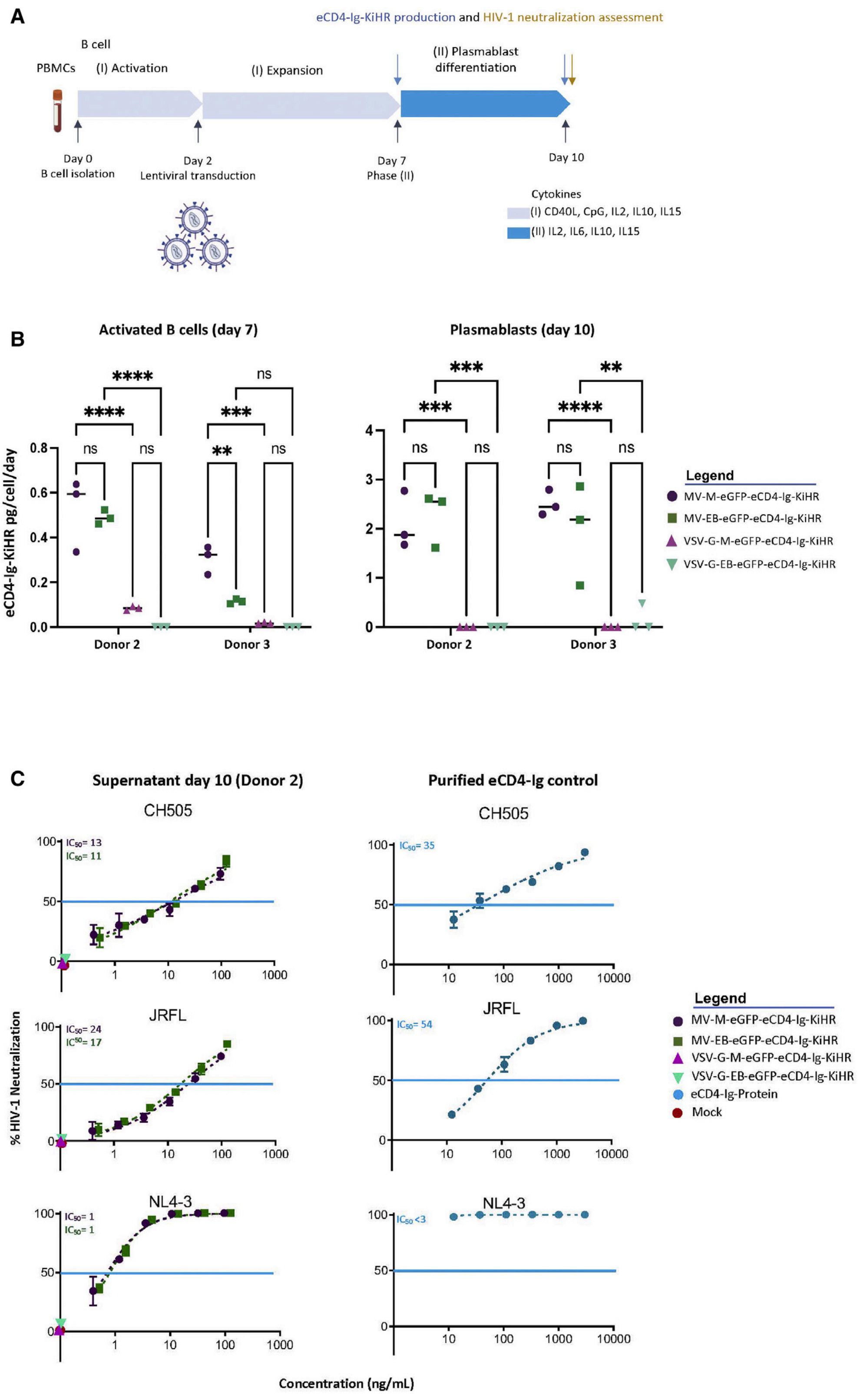

(legend on next page)

in plasmablast culture supernatants were similar regardless of whether ectopic TPST2 was provided (Figure 5C). A repeat experiment confirmed these findings (not shown). Finally, consistent with these findings, RNA sequencing revealed that TPST2 is expressed highly in human plasma cells (Figure 5D), which likely explains why ectopically expressed TPST2 did not further increase eCD4-Ig-KiHR activity in plasmablasts. Given the varied and essential cellular processes dependent on functional TPST2 expression,[71] overcoming the requirement for TPST2 transgene co-delivery simplifies vector design and potentially increases its safety profile, and further supports the LV platform for B cell eCD4-Ig-KiHR gene delivery.

MV-LV-mediated B cell transduction leads to high levels of eCD4-Ig secretion

To evaluate whether eCD4-Ig-KiHR expression changes as B cells differentiate into plasmablasts, we used a previously described two-step culture system[59] (Figure 6A). Primary B cells from two donors were transduced with relevant MV LV versus VSV-G LV viral constructs for comparison. Culture supernatants were collected from transduced, activated B cells (day 7) and plasmablasts (day 10), and eCD4-Ig-KiHR expression was assessed using an anti-gp140 ELISA immunoassay (Figure 4A). eCD4-Ig-KiHR expression was normalized for transduction efficiency, total number of B cells in culture, and phase duration (Figure 6A). First, eCD4-Ig-KiHR expression was minimal in cells transduced with the VSV-G pseudotyped LV, highlighting the need to achieve high transduction rates for substantive eCD4-Ig-KiHR expression levels. In contrast, MV LV transduction of B cells resulted in sustained eCD4-Ig-KiHR expression, particularly in plasmablasts (Figure 6B). Second, overall production of eCD4-Ig was substantially greater in plasmablasts than in activated B cells (Figure 6B). Finally, although expression of eCD4-Ig was higher at earlier time points with constructs expressing the MND promoter, there was little expression difference between the MND and EB promoters in plasmablasts, indicating that both promoters functioned effectively in regulating protein expression in LV transduced plasmablasts Figure 6B). These findings further support the use of the EmB29 promoter, given its high expression activity in B cell lineage and minimal expression activity in other cell types (Figure 1C and Sather et al.[48]).

We next assessed eCD4-Ig-KiHR HIV-1 neutralization activity from plasmablast supernatants (donor 2; day 10). As predicted by the poor transduction/expression levels (Figure 6B), supernatants from plasmablasts transduced with VSV-G-pseudotyped LV vectors exhibited no neutralization activity (Figure 6C). In contrast, B cell supernatants from MV-M-eGFP-eCD4-Ig-KiHR and MV-EB-eGFP-eCD4-Ig-KiHR transduced plasmablasts effectively, and supernatants from the stably transduced plasmablast neutralized all three HIV-1 isolates, NL4-3, JRFL, and CH505 (Figure 6C).

B cell eCD4-Ig-KiHR production prevents NL4-3 HIV-1 SupT1 $CD4^+$ T cell-to-T cell spread in a co-culture model

To examine the role of eCD4-Ig-KiHR and eCD4-Ig-WT to control a spreading T cell-to-T cell HIV-1 infection, we established co-culture model utilizing the Nalm6 B cells and NL4-3 HIV-1 infected SupT $CD4^+$ T cells. We utilized this cell-line-based model because attempts to utilize LV transduced primary B cells proved difficult because of poor viability when co-cultured with infected SupT1 T cells. To undertake the HIV-1 spreading study, stably transduced MV-M-eGFP-eCD4-Ig-KiHR and MV-EB-eGFP-eCD4-Ig-KiHR Nalm6 B cells producing eCD4-Ig-KiHR or the parental mock Nalm6 B cells were mixed with NL4-3 HIV-1-infected SupT1 $CD4^+$ T cells at two different T cell/B cell ratios and cultured for 6 days (Figure 7). As shown in Figure 7, there was HIV-1 replication in HIV-1-infected SupT1 $CD4^+$ T cells co-cultured with parental Nalm6 B cells at either T cell/B cell ratio by day 6 of co-culture (red line). In contrast, M-eGFP-eCD4-Ig-KiHR (gray line) and EB-eGFP-eCD4-Ig-KiHR (green line) Nalm6 B cells producing eCD4-Ig-KiHR suppressed HIV-1 spread 4-fold (1:10 T cell/B cell ratio) and 37-fold (1:4 T cell/B cell ratio) as judged by p24 measurements over time ($n = 4$, $p < 4 \times 10^{-4}$ at the 1:4 co-culture ratio and $p < 2 \times 10^{-6}$ 1:10 co-culture ratio). Anti-HIV-1 eCD4-Ig activity during T cell-to-T cell spread further supports that B cells, albeit a B cell line, have sufficient TPST2 to sulfonate eCD4-Ig to produce HIV-1 neutralization activity. Again, consistent with observation of Nalm6 B cells producing active eCD4-Ig-KiHR, RNA-sequencing studies have demonstrated the presence of TPST2 transcripts in Nalm6 cells.[72] Taken together, these combined results indicate that primary B cells produce eCD4-Ig-KiHR with HIV-1 neutralization activity and that a B cell line secreting eCD4-Ig-KiHR can effectively disrupt HIV-1 T cell-to-T cell spread.

DISCUSSION

B cells have appealing features for gene engineering to produce therapeutic proteins; they differentiate into long-lived memory and plasma cells capable of robust and long-term antibody production

Figure 6. MV LV B cell transduction of activated B cells results in eCD4-Ig production in activated B cells and plasmablasts and increasing production of eCD4-Ig in plasmablasts

(A) Schematic of the workflow of B cell studies performed. Supernatants were collected and assessed for eCD4-Ig-KiHR production on days 7 and 10 and for HIV-1 neutralization capacity on day 10. See materials and methods for culture condition information. B cells from donors 2 and 3. (B) eCD4-Ig-KiHR production from the listed (see legend) stably LV transduced B cells were normalized to the total number of live cells per day for each collection time. Data presented in dot plots are mean ± SD of triplicates. ns, not significant; **$p < 0.01$, ***$p < 0.01$, ****$p < 0.0001$. (C) eCD4-Ig-KiHR-containing supernatants from MV, but not VSV-G, pseudotyped LV transduced and differentiated plasmablasts can efficiently neutralize HIV-1 isolates. Supernatants were evaluated for eCD4-Ig-KiHR neutralization capacity from B cells. The neutralization assay, and HIV-1s used for evaluation, are described in Figure 4C. Paired one-sided Welch's t test on IC_{50} HIV-1 neutralization assessment of $n = 6$ eCD4-Ig-WT and $n = 6$ eCD4-Ig-KiHR repeats, three repeats for each LV promoter, $p < 0.022$. Blue horizontal line marks IC_{50}, and graph points show range and average of duplicates. For some samples, the lack of range is due to lack of variance between individual assays. IC_{50}s were calculated from six dilution points of each supernatant of interest.

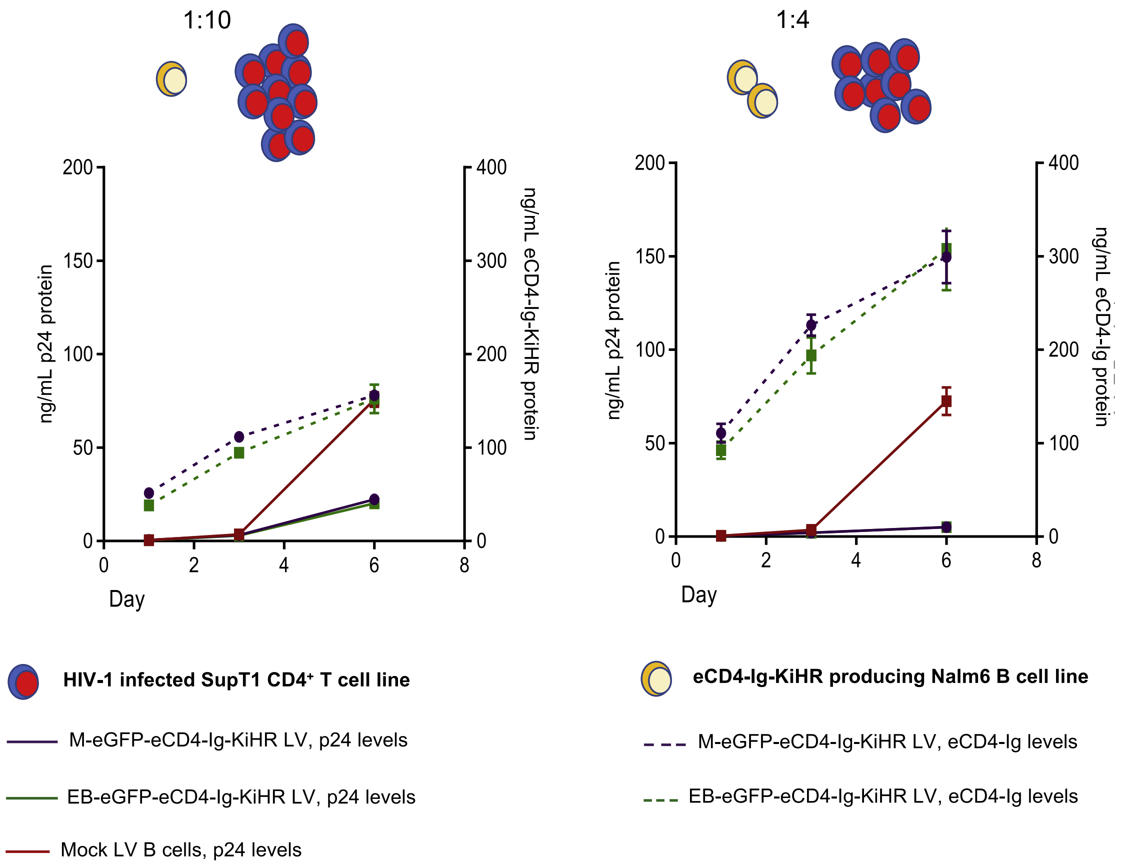

Figure 7. Nalm6 B cell line expression of eCD4-Ig-KiHR prevents NL4-3 HIV-1 spread in SupT1 CD4$^+$ T cells in a cell co-culture model of HIV-1 infection
Nalm6 B cells were transduced with either M-eGFP-eCD4-Ig-KiHR or EB-eGFP-eCD4-Ig-KiHR LVs and later mixed at a 1:10 or a 1:4 ratio of Nalm6 eCD4-Ig-expressing cells to NL4-3 HIV-1-infected SupT1 CD4$^+$ T cells. At the time of addition to Nalm6 B cells for co-culturing, SupT1 CD4$^+$ T cells were ~10% NL4-3 HIV-1 positive by p24 flow cytometry. Mock LV transduced Nalm6 cells (red lines) were used as controls. Cultures evaluated for HIV-1 p24 protein production (black solid lines, left axis) and eCD4-Ig production (dotted lines, right axis) on days 1, 3, and 6 post culture establishment. Dot plots indicate average protein production from four repeats, and error bars show SD. $p < 0.001$ for both co-culture ratios and for M and EB eCD4-Ig regulated LVs.

and migrate to lymphoid, bone marrow, and vascular compartments.[36,59,73] LV-based delivery, if made efficient via pseudotyping to target B cells,[44–47] is predicted to mediate sustained, high-level therapeutic protein expression and is capable of delivering a large therapeutic payload(s) to transduced primary B cells. As described previously, our findings demonstrate that VSV-G pseudotyped LV vectors mediate only minimal transduction of activated primary human B cells.[44] In contrast, using an optimized MV pseudotyped LV protocol improved LV transgene delivery to activated B cells by up to 75% frequency, levels higher than reported in previous studies involving alternative candidate LV pseudotypes.[44–47] Despite the length and complexity of the bicistronic vectors employed in this study, properties typically associated with decreased packaging and transduction performances,[74,75] the high-level B cell delivery observed likely reflects the impact of our optimized packaging and B cell transduction protocols.[60] Importantly, we observed consistent transduction rates across a range of human B cell donors, encouraging the pan-genetic and pan-gender use of this platform. In parallel with increased LV transduction, use of the EμB29 enhancer/promoter[48] permitted high-level B cell lineage expression at levels comparable with the robust MND gammaretroviral-derived promoter/enhancer. These finding were consistent with our previous studies in human and mouse B cell lines using LV-EμB29-eGFP, indicating the pan-species regulatory nature of EμB29.[48] Finally, robust eCD4-Ig-KiHR expression was achieved using the EμB29 enhancer/promoter with a clinically relevant VCN (~2.5), further supporting the notion that eCD4-Ig-KiHR production was the result of an efficient promoter/enhancer rather than multiple LV copies per B cell.

A major component of the protein-producing capacity of plasma cells is committed to Ig production.[37–39] Although this high capacity for protein expression in B cells provides a unique opportunity for therapeutic protein expression, including eCD4-Ig, endogenous IgG creates potential challenges, chiefly the potential for unwanted heterodimerization between synthetic and endogenous Fc domains. We used KiHR modifications of the CH3 Fc IgG domain of eCD4-Ig

that improved protein homodimerization, leading to improved functional protein expression in primary B cells. While efforts to optimize heterodimerization of Fc-containing proteins is of major therapeutic and commercial interest in the field of bispecific antibody production,[76] to our knowledge this study is the first to apply structure-based modifications to specifically disfavor heterodimerization between endogenous IgG and an ectopically expressed Fc-containing protein. Further molecular techniques from bispecific antibody development, such as Fc chain electrostatic optimization and other protein considerations, may have the potential to further improve expressed protein stability and functionality.[77] eCD4-Ig has been shown to promote ADCC activity, a trait uniquely useful in treating HIV-1 infection.[62] Bispecific IgGs designed with KiH mutations were previously shown to not alter ADCC functions.[78] Additionally, KiHR mutations in the Fc regions did not appear to overlap with mutations reported to affect FcR[79] or C1q binding.[80] These reports are consistent with predicted maintenance of ADCC effector functions in eCD4-Ig-KiHR, although this remains to be formally evaluated.

A second challenge specific to high-level, functional eCD4-Ig-KiHR expression is the requirement for post-translational tyrosine sulfation of the eCD4 co-receptor mimetic peptide for full anti-HIV-1 neutralization activity.[32,68] Unlike previous studies of eCD4-Ig production from non-human primate myocytes,[32] ectopic expression of TPST2 was not required in B cells. We observed equivalent eCD4-Ig-KiHR HIV-1 neutralization capacity in the presence or absence of *cis*-linked TPST2 expression. This observation correlated with our demonstration of high-level TPST2 mRNA expression in primary human plasma cells. This suggests that B cells, as specialized protein-secreting cells, may be better suited to produce soluble therapeutic proteins than previously studied systems. We (Figure 6E) and others show that TPST2, a protein with a key role in post-translational modification of secreted proteins and various cellular processes,[71,81] was highly expressed at the transcript level in differentiated B cells.[69] These findings permit a more simplified LV design for eCD4-Ig-KiHR delivery to primary B cells. Given that the physiological significance of TPST2 regulation is unclear,[49] the lack of ectopic TPST2 expression may also minimize unknown risks, permitting a safer delivery design. Our findings also suggest that gene delivery of other therapeutic proteins that require tyrosine sulfation for activity, such as Factors V and VIII, might similarly benefit from B cell expression.[71,82] Overall, given the specialized post-transcriptional and metabolic program of primary B cells, we anticipate that B cell gene delivery may provide an improved cellular environment for therapeutic protein expression and modification as compared with other cell types.

Traditional vaccines have so far failed to generate lasting protective antibody immunity against HIV-1.[83] HIV-1 bNAbs isolated from individuals have been shown to be effective initially when used as a monotherapy, but the virus generally becomes resistant.[24–26] Directed B cell immunoglobulin gene engineering to introduce novel anti-HIV paratropes has the distinct advantage that class switching and affinity maturation can occur, thereby potentially improving anti-HIV-1 breadth and neutralization potency.[24,25] However, as in vaccine development, there remains the high probability of HIV-1 resistance to highly effective bNAbs generated *in situ* from immunoglobulin gene engineering and serial immunization.

Important for the long-lasting efficiency of bNAbs in individuals is the ability to limit HIV escape. In most cases HIV-1 becomes resistant to the currently isolated bNAbs when used as single passive therapy treatments.[24,25] The studies published to date indicate that it remains to be determined what the ideal combination of epitope targets and/or the minimum required number of HIV-1 epitope specificities will be needed to avoid viral escape.[24,25] Interestingly, eCD4-Ig has the advantage of binding at high affinity to the HIV-1 envelope at both the CD4 and either co-receptor (X4- or R5-tropic) sites simultaneously, thus providing a higher genetic barrier for the virus to surmount to generate resistance.[33] In contrast to the majority of bNAbs,[24,25] HIV-1 resistance to eCD4-Ig extracts a considerable viral fitness cost, and eCD4-Ig still neutralizes viral escape variants to some degree.[33]

Our findings demonstrate that LV transduced B cells can express eCD4-Ig-KiHR at levels sufficient to disrupt T cell-to-T cell HIV spread *in vitro* (Figure 7). Importantly, for moving forward in evaluating whether modified B cells could provide sufficient eCD4-Ig-KiHR *in vivo* to control HIV-1 infection, culture supernatants demonstrated robust anti-HIV-1 activity against three HIV-1 isolates of both CXCR4 and CCR5 tropisms (Figures 5C and 6C). It is notable that B cell eCD4-Ig-KiHR production exhibits anti-HIV-1 potency within the ng/mL range (Figure 6C); in our *in vitro* cell culture system $\sim 10^6$ B cells were shown to express ~1 μg/mL in a total volume of 400 μL of culture, or ~5 pg/transduced cell/day, from EB regulated B cells (Figure 6B). Overall, our findings provide support for further evaluation of sustained levels of eCD4-Ig-KiHR and anti-HIV-1 responses in sera from LV EμB29-eGFP-eCD4-Ig-KiHR engineered B cells in appropriate humanized mouse models. Additional support for future engineered B cell studies in humanized mice comes from our studies showing that eCD4-Ig AAV-mediated direct delivery to non-human primate myocytes controls SHIV challenge for a time[32] and our recent report that *ex vivo* engineered human plasma cells home and function when transplanted to humanized mice.[36]

Finally, an open question in the field has been the concern of immune responses to *in vivo* transgene produced proteins, responses that can markedly reduce therapeutic effectiveness. Gene replacement therapies face the challenge of neoantigen introduction and subsequent recognition by cellular and humoral responses.[84] Development of anti-drug antibodies against administered therapeutic proteins is a well-documented complication[85,86] and a potential barrier to gene therapy treatments.[84,87,88] Of interest, reports have documented that gene products fused to IgG[89–91] and delivered to B cells have proved efficacious in inducing immunotolerance in various animal models.[89,92] These findings suggest that primary B cells might serve as a more appropriate target cell for the delivery of therapeutic transgenes. Future studies will be required to test this prediction in

appropriate *in vivo* models. Incorporation of tissue-specific promoters has also been shown to reduce potential immune responses to neo-transgene proteins,[84] as they regulate protein expression according to cell-specific control. Thus, use of the EμB29 promoter may further support a tolerogenic response to eCD4-Ig-KiHR *in vivo*.

Overall, our findings support further studies of B cell lineage expression of eCD4-Ig-KiHR as an approach for dampening HIV-1 infection. The use of LV-based B cell gene delivery and lineage-specific regulation may be useful for additional applications, ranging from anti-pathogen responses to therapeutic protein production *in vivo*.

MATERIALS AND METHODS

Plasmids

Promoters used for this study were introduced into the vector backbone using 5′*PacI* and 3′*BamHI* sites. The EμB29 promoter/enhancer was cloned from the pRRL-EμB29-eGFP209 plasmid. The eCD4-Ig fragment was generated from pCMV-eCD4-IgG1v34 with the introduction of a 3′*SbfI* site via PCR. pLV-EμB29-eGFP-eCD4-Ig and pLV-MND-eGFP-eCD4-Ig were generated by restriction fragment cloning into the pFG12-MND-eGFP-P2A-MCS LV backbone (courtesy of Kip Hermann, Bruce Torbett Lab, Scripps Research, La Jolla, CA). pFG12-MND-eGFP-P2A-MCS LV backbone was linearized via *SrfI* and *SbfI* digestion and ligated with the eCD4-Ig fragment above to generate pLV-MND-eGFP-P2A-eCD4-Ig. pLV-EμB29-eGFP-P2A-eCD4-Ig was generated by restriction fragment insertion of a fragment derived from pRRL-EμB29-eGFP with a 5′*XbaI* and 3′*PacI* site added via PCR. This fragment was ligated to the *XbaI* and *PacI* digested backbone fragment of pLV-MND-eGFP-P2A-eCD4-Ig to generate pLV-EμB29-eGFP-P2A-eCD4-Ig. The KiHR mutations were introduced into pLV-MND-eGFP-P2A-eCD4-Ig and pLV-EμB29-eGFP-P2A-eCD4-Ig using restriction fragment cloning. The IgG CH3 domains containing the KiHR T394F (ACA→TTC) and F405A (TTC→GCC) mutations were generated by gBlock fragment synthesis by Integrated DNA Technologies (Coralville, IA) and ligated with the *AleI* a *BamHI* digested backbones from pLV-MND-eGFP-P2A-eCD4-Ig and pLV-EμB29-eGFP-P2A-eCD4-Ig. TPST2-containing constructs were generated via PCR using pC3.1-TPST2c9 as template with introduction of a 5′*XhoI* and a 3′*BamHI* site. pLV-EμB29-TPST2-eCD4-Ig and pLV-MND-TPST2-eCD4-Ig were then generated by restriction fragment cloning using pLV-EμB29-eGFP-eCD4-Ig and pLV-MND-eGFP-eCD4-Ig as backbones. WT and KiHR variants were generated as detailed above. Measles Edmonston strain hemagglutinin (H) (GenBank: AB583749.1) and fusion117 (F) (GenBank: U03657.1) glycoprotein plasmids were a kind gift from Els Verhoeyen. Both glycoproteins were inserted into pCG plasmid driven by the cytomegalovirus early promoter (Addgene #51476). The cytoplasmic tail of the hemagglutinin and fusion glycoproteins were deleted by truncation of 24 and 30 amino acids, respectively, and have been described previously.[93] Generated plasmids were screened with both restriction digestion confirmation and Sanger sequencing to confirm successful cloning. All sequences and cloning strategies detailed above are available upon request.

Lentiviral vector production and titration

VSV-G pseudotyped lentiviral vector production

VSV-G pseudotyped LV was produced, stored, and titrated as previously described.[94]

Measles pseudotyped lentiviral vector production

A comprehensive description of all reagents and step-by-step protocols are discussed in Vamva et al.[60] CD46 KO 293T cells[94] were maintained in DMEM supplemented with 10% fetal bovine serum (FBS, Gibco) at 37°C. Cells (5×10^6) were seeded in a 0.01% poly-L-lysine-coated (Sigma-Aldrich) 10-cm^2 dish in a final volume of 10 mL. Eighteen hours later the medium was changed to promote cell proliferation. Two hours after medium change the cells were transfected using the Profection Mammalian transfection system (Promega) following the manufacturer's instructions. For each transfection plate, 500 mL of 2 M $CaCl_2$ containing a total of 28 μg of DNA, in a 1:1:1:1:4 M ratio of pMDLg/RRE (Addgene #12251)/pRSV-Rev (Addgene #12253)/Measles Edmonston strain H (GenBank: AB583749.1)/Measles Edmonston strain F (GenBank: U03657.1)/transfer vector were added dropwise to 500 mL of the 2× HEPES-buffered solution while bubbling air through it. Precipitates were allowed to form at room temperature for 30 min. The solution was then added dropwise across the plate, followed by swirling to ensure even distribution. Sixteen hours after transfection the medium was replaced with 4 mL of 3% FBS containing DMEM. Forty hours after transfection, supernatant was harvested and filtered through a 0.22-μm polyethersulfone filter (Millipore). For each transfer vector of interest, the supernatant from three transfection plates was pooled together (~12 mL) and mixed with 20% sucrose in Hank's balanced salt solution (Gibco). Measles pseudotyped LV was concentrated by ultracentrifugation at 19,400 rpm for 2 h 20 min. After ultracentrifugation the medium was decanted carefully, and the pellet was resuspended in Iscove's modified Dulbecco's medium (IMDM) at a 200-fold concentration and stored long term at −80°C.

Measles pseudotyped lentiviral vector functional titration

The functional titer of HF MV and VSV-G pseudotyped LV was determined by serial dilution on Nalm6 cells, by evaluation of eGFP expression 72 h post transduction, as previously described.[95] Nalm6 cells were seeded at 3×10^5 per well in a 6-well plate at a final volume of 2 mL of RPMI + 10% FBS (cRPMI). Unconcentrated HF MV pseudotyped supernatants were thawed and used to infect cells 18 h post seeding at a series of dilutions in a final volume of 500 μL of cRPMI containing 8 μg/mL Polybrene. Cells were assessed by flow cytometry for eGFP expression 48 h post transduction. Titers were calculated using the dilution condition in which the proportion of eGFP-positive cells was between 4% and 25%. The transduction efficiency of each LV was calculated by multiplying the number of seeded cells by the percentage of eGFP-positive cells and by the vector dilution factor, which was then divided by the total volume used for Nalm6 cell transduction per well. Transduction units (TU) were defined as the number of functional viral particles in the supernatant that could transduce a cell and were thus successfully expressing eGFP (TU/mL). TU/mL calculations were subsequently used to calculate MOI.

Blood donor information

Deidentified PBMCs were acquired under informed consent from the Fred Hutch Specimen Processing and Research Cell Bank (Protocol #3942) from consented donors and cryopreserved. Details of PBMC donors are as follows: Donor 1: male, 28 years old, white non-Hispanic/Latino. Donor 2: female, 24 years old, American/Indian/Alaskan native/white non-Hispanic/Latino. Donor 3: male, 26 years old, Hispanic or Latino.

Cord Blood $CD34^+$ cells were isolated from cord blood from deidentified samples obtained from the Cleveland Cord Blood Clinic (Cleveland, OH), under approved institutional protocols (IRB-17-6966 TSRI). $CD34^+$ cells were isolated as described in Ozog et al.[94]

Primary B cell isolation, transduction, and differentiation

PBMCs were collected from whole blood of consented donors and cryopreserved at the Fred Hutchinson Cancer Research Center. B cells were isolated by negative selection as per manufacturer's instructions (B cell isolation kit II, Miltenyi Biotec) and then cultured in IMDM supplemented with 10% FBS and 55 mM β-mercaptoethanol (aIMDM) at 1.5×10^6 cells/mL. B cells were activated with 100 ng/mL recombinant human MEGACD40L (Enzo Life Sciences), 1 μg/mL CpG oligodeoxynucleotide 2006 (Invitrogen), 50 ng/mL interleukin-2 (IL-2) (PeproTech), 50 ng/mL IL-10 (PeproTech), and 10 ng/mL IL-15 (PeproTech) (cIMDM) for 2 days, before transduction with LV. Cells (3×10^5) were seeded in a 96-well plate, mixed with 20 μL of measles pseudotyped (HF MV) LV, and spinoculated at 400 rpm for 30 min. Transduced cells were then expanded for 5 days in aIMDM before being differentiated using a previously described three-step culture system.[59] Cells were normalized to a 1.5×10^6 cells/mL concentration in IL-2 (50 ng/mL), IL-6 (50 ng/mL), IL-10 (50 ng/mL), and IL-15 (10 ng/mL) containing aIMDM for 3 days to induce plasmablast phenotype differentiation. Differentiation was confirmed by flow cytometry, detailed below.

Quantification of vector copy number by digital droplet PCR

B cells were harvested, and genomic DNA was isolated using the DNeasy Blood and Tissue kit (Qiagen). Approximately 25 ng of extracted genomic DNA was added to a 50-μL PCR reaction mix containing 2× digital droplet PCR (ddPCR) Supermix for Probes (Bio-Rad), a primer/probe set for the MKL2 reference gene and a primer/probe set for the target, which spans the psi element at a final concentration of 0.9 μM of each primer and 0.25 μM of each probe. Primer and probe sequences used are as follows. HIV-1 probe: 5′-FAM/AAA TCT CTA/ZEN/GCA GTG GCG CCC G/3IABkFQ-3′; HIV-1 forward (Fw) primer: 5′-CTG TTG TGT GAC TCT GGT AAC T-3′; HIV-1 reverse (Rv) primer: 5′-TTC GCT TTC AAG TCC CTG TT-3′; MKL2 probe: 5′-HEX/TGT TCC TGC/ZEN/AAC TGC AGA TCC TGA/3IABkFQ-3′; MKL2 Fw primer: 5′-AGA TCA GAA GGG TGA GAA GAA TG-3′; MKL2 Rv primer: 5′-GGA TGG TCT GGT AGT TGT AGT G-3′. Technical duplicates of 20 μL of the final mix were added to a 96-well plate, and droplets were generated using the Automated Droplet Generator (Bio-Rad) following the manufacturer's instructions. PCR was performed using the following thermal cycling conditions: 95°C for 10 min, 94°C for 30 s, and 60°C for 1 min (39 cycles), 98°C for 10 min (1 cycle), and 12°C hold. Droplets were read using a QX100 droplet reader, and data were analyzed using the QuantaSoft software suite to calculate average VCN per cell.

Quantification of vector copy number by qPCR

Total DNA was extracted from frozen pelleted $CD34^+$ cells using the DNeasy Blood and Tissue kit (Qiagen #69506). Pellets were treated with *DpnI* (New England Biolabs) for 2 h to ensure elimination of any residual packaging plasmid DNA. qPCR was carried out according to a previously published protocol using the probes specific to the integrated U5 regions of the HIV genome (primers MH531, MH532, LTR-P).[96] Reactions were run on a Roche LightCycler 480, using LightCycler 480 Probes Master (Roche) as previously described.[96]

Protein quantification with ELISA

Lentiviral vector physical titer

ELISA p24 was performed to measure p24-Capsid protein concentration and, thus, Gag protein expression, which is associated with physical LV titers. Viral supernatant was analyzed for Gag expression using the Lenti-X p24 Rapid Titer ELISA Kit (Takara) as per manufacturer's instructions.

eCD4-Ig quantification

Supernatant from transduced B cells was harvested 5 days, 8 days, and 11 days post transduction to study ecCD4-Ig expression, while supernatant from transduced Nalm6 cells was harvested 7 days post transduction. Cell supernatant was analyzed for eCD4-Ig expression using an in-house ELISA assay. HIV-1 BG505-derived gp-140 NFL trimer purified protein (NIH AIDS Reagent Bank, #13418) was diluted in 100 mM $NaHCO_3$ (pH 8.5) to 2.5 μg/mL to coat high binding ELISA plates (Corning, Costar) overnight at 4°C. The next day, plates were automatedly washed six times with PBS containing 0.05% Tween 20 using the 50 TS microplate washer (BioTek). Wells were subsequently blocked with 3% BSA in PBS for 1 h at room temperature and then washed as described above. eCD4-Ig containing cell supernatant was diluted in 1% BSA in PBS and added to wells for 1 h at room temperature. The wells were then washed again as described above and subsequently incubated for 1 h at room temperature with Peroxidase AffiniPure goat anti-human IgG (H + L) (Jackson ImmunoResearch Laboratories, #109-035-088) diluted 1:10,000 in 1% BSA PBS. After a final washing step, samples were developed in 1-step Ultra TMB-ELISA substrate (Pierce, #34028) for 10 min and quenched with 0.5 M H_2SO_4. Absorbance was read at 450 nM on a BioTek μQuant Universal microplate spectrophotometer. eCD4-Ig quantification was calculated using a 4-parameter calculated standard curve generated by serial dilution of eCD4-Ig purified protein isolated by protein A purification via high-performance liquid chromatography.

IgG quantification

Quantitative detection of human IgG from B cell supernatant was achieved using the commercially available Invitrogen (Total) Human

ELISA Kit (Fischer Scientific, #88-50550) following the manufacturer's instructions. Supernatant from transduced B cells was analyzed at 5 days, 8 days, and 11 days post transduction. Optimal detection and quantification were achieved when B cell supernatants were diluted 1:250 in assay buffer A.

Dimerization studies

eCD4-Ig- and VRCO1-containing supernatants were produced by polyethyleneimine (PEI)-mediated (Polysciences) transient co-transfections in HEK293T cells. In brief, 5×10^6 cells were plated on poly-L-lysine-coated 10-cm^2 tissue culture treated Petri dishes in 10% FBS containing DMEM. After 24 h, medium was exchanged for DMEM containing 3% FBS before addition of plasmids. Branched PEI was mixed with 7.5 μg/plate of the pCMV-eCD4Ig-WT, pCMVeCD4-Ig-KiHR and 22.5 μL of 1 mg/mL in 1 mL of OptiMEM (Gibco, Life Technologies, Waltham, MA). For co-transfection studies, 2.5 μg/plate each of pCMV-VRCO1 HC and pCMV-VRCO1 LC were mixed with 2.5 μg/plate of the above CMV-eCD4-Ig constructs. The DNA/PEI mix was then thoroughly mixed and incubated for 15 min before being added dropwise on seeded cells. Supernatant from co-transfections was harvested 48 h post transfection. eCD4-Ig- and VRCO1-containing cell supernatants were measured for protein concentration with the BCA Protein Assay kit (Pierce, #23227) and normalized for protein loading. Samples were run on a 4%–12% Bis-Tris Bolt Precast Gel (Thermo Fisher, #NW04125BOX) under non-reducing conditions and transferred to an activated polyvinylidene fluoride (PVDF)-FL membrane. Total protein on membrane was visualized by Revert Total Protein Stain (Licor, #926-11010). Membranes were blocked in 3% milk in PBS for 1 h at room temperature then incubated with Peroxidase AffiniPure goat anti-human IgG (H + L) diluted 1:10,000 in 1% milk in PBS. Blots were imaged using ECL Western Blotting Substrate (Pierce, #32106) and visualized on the Azure c600 imaging system (Azure Biosystems).

Western blotting

IgG Fc detection

Supernatant from transduced B cells was harvested 5 days, 8 days, and 11 days post transduction to study eCD4-Ig expression and dimerization status at the different stages of B cell differentiation. Equal volumes of eCD4-Ig-containing cell supernatants were mixed with cold 4× Bolt LDS Sample Buffer (Thermo Fisher) and directly loaded on a 4%–12% Bis-Tris Bolt Precast Gel (Thermo Fisher) in cold 1× Bolt MES SDS running under non-reducing conditions to preserve protein dimers. The gel was then transferred to an activated PVDF membrane using the Trans-Blot turbo transfer system (Bio-Rad). Membranes were blocked in 5% milk in PBS for 2 h at room temperature and were subsequently incubated with Peroxidase AffiniPure goat anti-human IgG (H + L) (Jackson ImmunoResearch Laboratories, #109-035-088) diluted 1:10,000 in 2% milk in PBS for 2 h at room temperature. Blots were washed in 0.1% Tween containing 1× Tris-buffered saline (TBST) and imaged using ECL Western Blotting Substrate. Results were visualized on the Azure c600 imaging system (Azure Biosystems).

CD4 detection

Sample preparation, loading, membrane transfer, and blocking were performed as described above. Membranes were blocked in 5% milk in PBS and then incubated with the recombinant anti-CD4 antibody (Abcam, #ab133616) diluted 1:1,000 in 2% milk in PBS overnight at 4°C. Blots were then washed in 0.1% TBST and incubated with Peroxidase AffiniPure goat anti-human IgG (H + L) (Jackson ImmunoResearch Laboratories, #109-035-088) diluted 1:10,000 in 5% BSA in TBS for 2 h at room temperature. Blots were washed, imaged, and visualized as described above.

Sulfotyrosine detection

HEK293T cells (5×10^6) were seeded in a 10-cm^2 dish in a final volume of 10 mL of cDMEM. After 18 h the medium was changed to promote cell proliferation. Two hours after medium change the cells were transfected using the Profection Mammalian transfection system (Promega) following the manufacturer's instructions. For each transfection plate, 500 mL of 2 M $CaCl_2$ containing a total of 15 μg DNA of either MND-eGFP-eCD4-Ig-KiHR or MND-TPST2-eCD4-Ig-KiHR transfer vector plasmids were added dropwise to 500 mL of the 2× HEPES-buffered solution while bubbling air through it. Precipitates were allowed to form at room temperature for 30 min. The solution was then added dropwise across the plate, followed by swirling to ensure even distribution. Sixteen hours after transfection the medium was replaced with 5 mL of 3% FBS containing DMEM. Seventy-two hours after medium change, eGFP-eCD4-Ig-KiHR and TPST2-eCD4-Ig-KiHR eCD4-Ig-containing supernatants were measured for eCD4-Ig-KiHR concentration with the anti-eCD4 ELISA as described above. Twenty micrograms of eCD4-Ig-KiHR, expressed by either the MND-eGFP-eCD4-Ig-KiHR or the MND-TPST2-eCD4-Ig-KiHR transfer vector plasmid, was loaded on a 4%–12% Bis-Tris Bolt Precast Gel (Thermo Fisher, #NW04125BOX) under non-reducing conditions, electrophoresed, and transferred to an activated PVDF-FL membrane. Membranes were blocked in 3% milk in PBS for 1 h at room temperature. The presence of eCD4-Ig sulfonated tyrosines was investigated with the use of the anti-sulfotyrosine antibody (clone Sulfo-1c-A2, Millipore).

Flow cytometry

Flow-cytometry analysis was performed on a BD Biosciences LSR-II cytometer equipped with UV, violet, blue, and red lasers. Doublets and dead cells were excluded by forward scatter, side scatter, and use of the AF350 LIVE/DEAD fixable violet dead cell stain (Thermo Fisher, #L34964, 1:1000). B cells were tested for CD19 (BioLegend, #302243 Brilliant Violet 605 anti-human CD19 antibody, 1:200), CD3 (BioLegend, #317329, Brilliant Violet 785 anti-human CD3 antibody, 1:200), and CD38 (BD, #551400, PerCP-Cy5.5 mouse anti-human CD38, 1:200). SupT1 cells were tested for CD8 (BD, #560662, PerCP-Cy5.5 mouse anti-human CD8, 1:100). Cells were fixed with the Cytofix/Cytopermreagent and washed in Perm/Wash buffer (BD). Cells were then permeabilized using Permeabilization Plus Buffer (BD, #561651). B cells were subsequently stained intracellularly for IgG (BioLegend, #410707, PE anti-human IgG Fc antibody, 1:200) and IgM (BioLegend, #314532PE/Cyanine7 anti-human IgM

antibody, 1:200), and SupT1 cells were tested for p24 (Beckman Coulter, #6604667, HIV-1 core antigen-RD1, KC57, 1:100).

HIV-1 neutralization assays

HIV *env*s JRFL and NL4-3 in the pSVIII expression plasmid, as well as the *env*-deleted backbone plasmid pSG3ΔEnv, were obtained from the NIH HIV Reagent Program. CH505 *env*[97] was synthesized (GenScript) and cloned into the pLenti-III plasmid. HIV pseudotyped virus was produced by transient co-transfection of HEK293T cells with *env* plasmid DNA and pSG3ΔEnv using 25 kDa PEI (Polysciences) as previously described.[98] HIV-1 neutralization assays were performed as previously described,[67] with slight modifications. In brief, TZM-bl target cells were seeded onto half-well 96-well white plates in 50 μL of growth medium (DMEM containing 10% FBS, 20 mM L-glutamine, 100 U/mL penicillin, and 100 μg/mL streptomycin) at 1×10^5 cells/mL and incubated overnight at 37°C. Pseudotyped viruses (24 μL) were co-incubated with a dilution series of B cell supernatant or monoclonal antibody (8 μL) at 37°C for 1 h. The maximum possible volume, 25% of total volume, was used for the mock samples. Subsequently, medium was aspirated from the TZM-bl cells and 25 μL of the virus/supernatant mixture was added. The plates were incubated at 37°C for 16 h, then 75 μL of fresh medium was added to minimize evaporation. Infectivity was determined 72 h post infection by lysing the cells, adding Bright-Glo Reagent (Promega), and measuring luciferase activity with a Synergy H1 plate reader (BioTek). HIV-1 strains used: NL4-3 (CXCR-4 tropic, clade B, tier 1A, T cell line adapted strain), JRFL (CCR5 tropic, clade B, tier 2 isolate), and CH505 (CCR5 tropic, clade C, tier 2 transmitted founder primary isolate).

T cell and B cell co-culture HIV-1 spread assay

Nalm6 cells were transduced with MND-eGFP-eCD4-Ig-KiHR and EμB29-eGFP-eCD4-Ig-KiHR LVs and subsequently tested for VCN to yield about one integrated copy per cell. In brief, 10^5 Nalm6 cells were incubated with serial dilutions of LV matched for input p24 protein for 24 h before wash and expansion for an additional 3 days. VCN was determined by qPCR according to a previously published protocol with probes specific to integrated U5 to J regions of the HIV genome (primers MH531, MH532, LTR-P).[96] Cells with VCN of ∼1 were cultured for 2 weeks to ensure stable eCD4-Ig-KiHR production. Low-passage SupT1 cells, 6×10^6 per well, were infected with 1 μg of p24 protein equivalent of molecular clone NL4-3 HIV-1 in complete RPMI 1640 medium at 10^6 cells/mL. Cells were incubated for 12 h at 37°C in a 5% CO_2 incubator before pelleting at $300 \times g$ for 5 min and washing three times in fresh RPMI medium to remove bound virus. Infected cells (10^5) were taken at this point and tested for intracellular p24 protein production. Cells were fixed/permeabilized with Cytofix/CytoPerm reagent and washed in Perm/Wash buffer as above. Cells were pelleted and resuspended in anti-p24 anti-HIV-1 core antigen-RD1 and analyzed as described in the flow cytometry section. Infected SupT1 cells (2.5×10^5 cells per well) were then plated in a 48-well plate with an equivalent number of uninfected SupT1 cells, and either 6.25×10^4 (1:4 ratio) or 2.5×10^4 (1:10 ratio) eCD4-Ig-KiHR producing Nalm6 cells. Cells were cultured for 6 days with counting and collection of supernatants on days 1, 3, and 6 post culture establishment, maintaining cell concentration at 10^6 cells/mL, for evaluation of p24 protein and eCD4-Ig production by respective ELISA immunoassays.

RNA sequencing

Following *ex vivo* differentiation of activated human primary B cells, cells were isolated using the EasySep Human CD138 positive isolation kit. Plasmablasts were isolated from the flow-through using positive selection with a CD38 MicroBead Kit (Miltenyi Biotec). Isolated plasmablasts were stored in TRIzol at −80°C. RNA extraction was performed using the RNeasy mini kit (Qiagen). cDNA synthesis, library preparation, and RNA sequencing were then performed in the Benaroya Research Institute Genomics Core Lab using Clontech's SMARTseq and Illumina's NexteraXT kits. Generated FASTQ files were filtered, and adaptors were trimmed using Trimmomatic. STAR was subsequently used to map high-quality reads to the human genome reference sequence (version hg38) and associate read counts with each gene.[99] A waterfall plot was created using the following python script GitHub: https://github.com/rene2718/EIRINI_rnaseq/blob/main/waterfall.ipynb.

Statistical analysis

Statistical analysis was performed by running relevant statistical models in R.[100] A linear mixed-effect model (LMM) was fitted to regress transduction efficiency against promoter (MND or EμB29), B cell status, and cell type, with included random promoter slopes for each cell type (REH, Nalm6, Ramos, Raji, SupT1, THP-1, $CD34^+$). A two-sided t test was performed to compare transduction efficiency differences between the two different promoters depending on their B cell status. Similarly, an LMM was fitted to regress transduction efficiency and VCN against promoter (MND or EuB29) with included random intercepts and promoter slopes for each environment type (DMSO, cara, PGE-2, cara + PGE-2). Two-sided t tests were performed to compare transduction efficiency and VCN for the two promoters under investigation. Both a paired one-sided Welch's t test and a paired one-sided Wilcoxon signed-rank test was run on IC_{50} measurements of WT and KiHR eCD4-Ig variants to assess the potential effect of mutations on eCD4-Ig neutralization potency. LMMs followed by two-sided t tests were employed to investigate the effect of envelope and promoter on transduction efficiency and protein expression over three differentiation phases for two independent donors. LMMs were fitted to regress number of transduced cells and protein expression levels against promoter (MND or EμB29) and envelope (MV and VSV-G) with included random intercept terms for donors 2 and 3. Two-sided Welch's two-sample t tests were performed to test a difference in p24 detection means between the SupT1 group treated with mock Nalm6 cells and the SupT1 groups treated with transduced Nalm6 cells. Statistical findings from these co-culture experiments were also confirmed with pairwise Wilcoxon rank sum tests and a non-parametric exact test of a difference in distributions, with Bonferroni correction for multiple testing.

Structural modeling

IgG1-Fc models were generated in UCSF Chimera, as modifications from PDB: 10.2210/pdb1H3X/pdb.

DATA AVAILABILITY

If materials are not available commercially or from Addgene, the materials presented here are available upon request after the execution of an MTA with Scripps Research Insitute or Seattle Children's Research Institute. Data are available upon request.

SUPPLEMENTAL INFORMATION

Supplemental information can be found online at https://doi.org/10.1016/j.omtm.2023.02.004.

ACKNOWLEDGMENTS

These studies were supported by the following awards: NIH/NIAID U54AI150472 (to B.E.T.), the Bill and Melinda Gates Foundation INV-017542 (to B.E.T., R.G.J., and D.J.R.), and NIH/NIAID AI143563 and AI152523 (to M.B.Z.). E.V. was supported by the Bill and Melinda Gates Foundation, S.O. was supported by a fellowship from the NIH/NHLBI, F30HL137563.

AUTHOR CONTRIBUTIONS

E.V. and S.O. performed most of the experiments. E.V., S.O., and B.E.T. analyzed the data and created the figures. E.V. wrote a draft which was extensively modified by feedback and contributions from S.O., M.B.Z., R.G.J., D.J.R., and B.E.T.and B.E.T. performed final manuscript revisions.D.P.L. performed the neutralization assays. R.Y.-H.C. performed the RNA-sequencing analysis in plasma cells. N.J.I. performed the statistical analysis under supervision of E.V. S.O., M.F., M.R.G., R.G.J., D.J.R., and B.E.T. conceptualized the study. S.O., E.V., and B.E.T. designed the study. B.E.T., R.G.J., and D.J.R. acquired funding for the study. All authors edited and approved the document.

DECLARATION OF INTERESTS

The authors declare no competing interests.

REFERENCES

1. HIV.gov/hiv-basics/overview/data-and-trends/global-statistics (2021). HIV global statistics.
2. Kiem, H.P., Jerome, K.R., Deeks, S.G., and McCune, J.M. (2012). Hematopoietic-stem-cell-based gene therapy for HIV disease. Cell Stem Cell *10*, 137–147.
3. Stan, R., and Zaia, J.A. (2014). Practical considerations in gene therapy for HIV cure. Current HIV/AIDS reports *11*, 11–19.
4. Swan, C.H., Buhler, B., Steinberger, P., Tschan, M.P., Barbas, C.F., 3rd, and Torbett, B.E. (2006). T-cell protection and enrichment through lentiviral CCR5 intrabody gene delivery. Gene Ther. *13*, 1480–1492.
5. Yuan, J., Wang, J., Crain, K., Fearns, C., Kim, K.A., Hua, K.L., Gregory, P.D., Holmes, M.C., and Torbett, B.E. (2012). Zinc-finger nuclease editing of human cxcr4 promotes HIV-1 CD4(+) T cell resistance and enrichment. Mol. Ther. *20*, 849–859.
6. Holt, N., Wang, J., Kim, K., Friedman, G., Wang, X., Taupin, V., Crooks, G.M., Kohn, D.B., Gregory, P.D., Holmes, M.C., et al. (2010). Human hematopoietic stem/progenitor cells modified by zinc-finger nucleases targeted to CCR5 control HIV-1 in vivo. Nat. Biotechnol. *28*, 839–847.
7. Younan, P.M., Polacino, P., Kowalski, J.P., Peterson, C.W., Maurice, N.J., Williams, N.P., Ho, O., Trobridge, G.D., Von Laer, D., Prlic, M., et al. (2013). Positive selection of mC46-expressing CD4+ T cells and maintenance of virus specific immunity in a primate AIDS model. Blood *122*, 179–187.
8. Falkenhagen, A., and Joshi, S. (2018). Genetic strategies for HIV treatment and prevention. Mol. Ther. Nucleic Acids *13*, 514–533.
9. Wang, C.X., and Cannon, P.M. (2016). The clinical applications of genome editing in HIV. Blood *127*, 2546–2552.
10. Balazs, A.B., Chen, J., Hong, C.M., Rao, D.S., Yang, L., and Baltimore, D. (2011). Antibody-based protection against HIV infection by vectored immunoprophylaxis. Nature *481*, 81–84.
11. Casazza, J.P., Cale, E.M., Narpala, S., Yamshchikov, G.V., Coates, E.E., Hendel, C.S., Novik, L., Holman, L.A., Widge, A.T., Apte, P., et al. (2022). Safety and tolerability of AAV8 delivery of a broadly neutralizing antibody in adults living with HIV: a phase 1, dose-escalation trial. Nat. Med. *28*, 1022–1030.
12. Caskey, M., Klein, F., Lorenzi, J.C., Seaman, M.S., West, A.P., Jr., Buckley, N., Kremer, G., Nogueira, L., Braunschweig, M., Scheid, J.F., et al. (2015). Viraemia suppressed in HIV-1-infected humans by broadly neutralizing antibody 3BNC117. Nature *522*, 487–491.
13. Fuchs, S.P., Martinez-Navio, J.M., Piatak, M., Jr., Lifson, J.D., Gao, G., and Desrosiers, R.C. (2015). AAV-delivered antibody mediates significant protective effects against SIVmac239 challenge in the absence of neutralizing activity. PLoS Pathog. *11*, e1005090.
14. Brady, J.M., Phelps, M., MacDonald, S.W., Lam, E.C., Nitido, A., Parsons, D., Boutros, C.L., Deal, C.E., Garcia-Beltran, W.F., Tanno, S., et al. (2022). Antibody-mediated prevention of vaginal HIV transmission is dictated by IgG subclass in humanized mice. Sci. Transl. Med. *14*, eabn9662.
15. Martinez-Navio, J.M., Fuchs, S.P., Pantry, S.N., Lauer, W.A., Duggan, N.N., Keele, B.F., Rakasz, E.G., Gao, G., Lifson, J.D., and Desrosiers, R.C. (2019). Adeno-associated virus delivery of anti-HIV monoclonal antibodies can drive long-term virologic suppression. Immunity *50*, 567–575.e565.
16. Kuhlmann, A.S., Haworth, K.G., Barber-Axthelm, I.M., Ironside, C., Giese, M.A., Peterson, C.W., and Kiem, H.P. (2019). Long-term persistence of anti-HIV broadly neutralizing antibody-secreting hematopoietic cells in humanized mice. Mol. Ther. *27*, 164–177.
17. Johnson, P.R., Schnepp, B.C., Zhang, J., Connell, M.J., Greene, S.M., Yuste, E., Desrosiers, R.C., and Clark, K.R. (2009). Vector-mediated gene transfer engenders long-lived neutralizing activity and protection against SIV infection in monkeys. Nat. Med. *15*, 901–906.
18. Lewis, A.D., Chen, R., Montefiori, D.C., Johnson, P.R., and Clark, K.R. (2002). Generation of neutralizing activity against human immunodeficiency virus type 1 in serum by antibody gene transfer. J. Virol. *76*, 8769–8775.
19. Priddy, F.H., Lewis, D.J.M., Gelderblom, H.C., Hassanin, H., Streatfield, C., LaBranche, C., Hare, J., Cox, J.H., Dally, L., Bendel, D., et al. (2019). Adeno-associated virus vectored immunoprophylaxis to prevent HIV in healthy adults: a phase 1 randomised controlled trial. Lancet HIV *6*, e230–e239.
20. Diskin, R., Scheid, J.F., Marcovecchio, P.M., West, A.P., Jr., Klein, F., Gao, H., Gnanapragasam, P.N., Abadir, A., Seaman, M.S., Nussenzweig, M.C., et al. (2011). Increasing the potency and breadth of an HIV antibody by using structure-based rational design. Science *334*, 1289–1293.
21. Huang, J., Ofek, G., Laub, L., Louder, M.K., Doria-Rose, N.A., Longo, N.S., Imamichi, H., Bailer, R.T., Chakrabarti, B., Sharma, S.K., et al. (2012). Broad and potent neutralization of HIV-1 by a gp41-specific human antibody. Nature *491*, 406–412.
22. Walker, L.M., Huber, M., Doores, K.J., Falkowska, E., Pejchal, R., Julien, J.P., Wang, S.K., Ramos, A., Chan-Hui, P.Y., Moyle, M., et al. (2011). Broad neutralization coverage of HIV by multiple highly potent antibodies. Nature *477*, 466–470.
23. Scheid, J.F., Mouquet, H., Ueberheide, B., Diskin, R., Klein, F., Oliveira, T.Y., Pietzsch, J., Fenyo, D., Abadir, A., Velinzon, K., et al. (2011). Sequence and structural convergence of broad and potent HIV antibodies that mimic CD4 binding. Science *333*, 1633–1637.
24. Julg, B., and Barouch, D. (2021). Broadly neutralizing antibodies for HIV-1 prevention and therapy. Semin. Immunol. *51*, 101475.

25. Sok, D., and Burton, D.R. (2018). Recent progress in broadly neutralizing antibodies to HIV. Nat. Immunol. *19*, 1179–1188.

26. Walsh, S.R., and Seaman, M.S. (2021). Broadly neutralizing antibodies for HIV-1 prevention. Front. Immunol. *12*, 712122.

27. Voss, J.E., Gonzalez-Martin, A., Andrabi, R., Fuller, R.P., Murrell, B., McCoy, L.E., Porter, K., Huang, D., Li, W., Sok, D., et al. (2019). Reprogramming the antigen specificity of B cells using genome-editing technologies. Elife *8*.

28. Nahmad, A.D., Lazzarotto, C.R., Zelikson, N., Kustin, T., Tenuta, M., Huang, D., Reuveni, I., Nataf, D., Raviv, Y., Horovitz-Fried, M., et al. (2022). In vivo engineered B cells secrete high titers of broadly neutralizing anti-HIV antibodies in mice. Nat. Biotechnol. *40*, 1241–1249.

29. Capon, D.J., Chamow, S.M., Mordenti, J., Marsters, S.A., Gregory, T., Mitsuya, H., Byrn, R.A., Lucas, C., Wurm, F.M., et al. (1989). Designing CD4 immunoadhesins for AIDS therapy. Nature *337*, 525–531.

30. Ashkenazi, A., and Chamow, S.M. (1995). Immunoadhesins: an alternative to human monoclonal antibodies. Methods *8*, 104–115.

31. Chiang, J.J., Gardner, M.R., Quinlan, B.D., Dorfman, T., Choe, H., and Farzan, M. (2012). Enhanced recognition and neutralization of HIV-1 by antibody-derived CCR5-mimetic peptide variants. J. Virol. *86*, 12417–12421.

32. Gardner, M.R., Kattenhorn, L.M., Kondur, H.R., von Schaewen, M., Dorfman, T., Chiang, J.J., Haworth, K.G., Decker, J.M., Alpert, M.D., Bailey, C.C., et al. (2015). AAV-expressed eCD4-Ig provides durable protection from multiple SHIV challenges. Nature *519*, 87–91.

33. Fellinger, C.H., Gardner, M.R., Weber, J.A., Alfant, B., Zhou, A.S., and Farzan, M. (2019). eCD4-Ig limits HIV-1 escape more effectively than CD4-ig or a broadly neutralizing antibody. J. Virol. *93*.

34. Gardner, M.R., Fellinger, C.H., Kattenhorn, L.M., Davis-Gardner, M.E., Weber, J.A., Alfant, B., Zhou, A.S., Prasad, N.R., Kondur, H.R., Newton, W.A., et al. (2019). AAV-delivered eCD4-Ig protects rhesus macaques from high-dose SIVmac239 challenges. Sci. Transl. Med. *11*.

35. Jeske, A.M., Boucher, P., Curiel, D.T., and Voss, J.E. (2021). Vector strategies to actualize B cell–based gene therapies. J. Immunol. *207*, 755–764.

36. Cheng, R.Y., Hung, K.L., Zhang, T., Stoffers, C.M., Ott, A.R., Suchland, E.R., Camp, N.D., Khan, I.F., Singh, S., Yang, Y.J., et al. (2022). Ex vivo engineered human plasma cells exhibit robust protein secretion and long-term engraftment in vivo. Nat. Commun. *13*, 6110.

37. Radbruch, A., Muehlinghaus, G., Luger, E.O., Inamine, A., Smith, K.G., Dörner, T., and Hiepe, F. (2006). Competence and competition: the challenge of becoming a long-lived plasma cell. Nat. Rev. Immunol. *6*, 741–750.

38. Slifka, M.K., Antia, R., Whitmire, J.K., and Ahmed, R. (1998). Humoral immunity due to long-lived plasma cells. Immunity *8*, 363–372.

39. Nutt, S.L., Hodgkin, P.D., Tarlinton, D.M., and Corcoran, L.M. (2015). The generation of antibody-secreting plasma cells. Nat. Rev. Immunol. *15*, 160–171.

40. Castiglioni, P., Gerloni, M., and Zanetti, M. (2004). Genetically programmed B lymphocytes are highly efficient in inducing anti-virus protective immunity mediated by central memory CD8 T cells. Vaccine *23*, 699–708.

41. Serafini, M., Naldini, L., and Introna, M. (2004). Molecular evidence of inefficient transduction of proliferating human B lymphocytes by VSV-pseudotyped HIV-1-derived lentivectors. Virology *325*, 413–424.

42. Brendel, C., Rio, P., and Verhoeyen, E. (2020). Humanized mice are precious tools for evaluation of hematopoietic gene therapies and preclinical modeling to move towards a clinical trial. Bio. Pharm. *174*, 113711.

43. Milone, M.C., and O'Doherty, U. (2018). Clinical use of lentiviral vectors. Leukemia *32*, 1529–1541.

44. Frecha, C., Costa, C., Lévy, C., Nègre, D., Russell, S.J., Maisner, A., Salles, G., Peng, K.-W., Cosset, F.-L., and Verhoeyen, E. (2009). Efficient and stable transduction of resting B lymphocytes and primary chronic lymphocyte leukemia cells using measles virus gp displaying lentiviral vectors. Blood *114*, 3173–3180.

45. Lévy, C., Amirache, F., Costa, C., Frecha, C., Muller, C.P., Kweder, H., Buckland, R., Cosset, F.-L., and Verhoeyen, E. (2012). Lentiviral vectors displaying modified measles virus gp overcome pre-existing immunity in in vivo-like transduction of human T and B cells. Mol. Ther. *20*, 1699–1712.

46. Levy, C., Fusil, F., Amirache, F., Costa, C., Girard-Gagnepain, A., Negre, D., Bernadin, O., Garaulet, G., Rodriguez, A., Nair, N., et al. (2016). Baboon envelope pseudotyped lentiviral vectors efficiently transduce human B cells and allow active factor IX B cell secretion in vivo in NOD/SCIDγc(-/-) mice. J Thromb Haemost *14*, 2478–2492.

47. Mock, U., Thiele, R., Uhde, A., Fehse, B., and Horn, S. (2012). Efficient lentiviral transduction and transgene expression in primary human B cells. Hum. Gene Ther. Methods *23*, 408–415.

48. Sather, B.D., Ryu, B.Y., Stirling, B.V., Garibov, M., Kerns, H.M., Humblet-Baron, S., Astrakhan, A., and Rawlings, D.J. (2011). Development of B-lineage predominant lentiviral vectors for use in genetic therapies for B cell disorders. Mol. Ther. *19*, 515–525.

49. Moore, K.L. (2003). The biology and enzymology of protein tyrosine O-sulfation. J. Biol. Chem. *278*, 24243–24246.

50. Astrakhan, A., Sather, B.D., Ryu, B.Y., Khim, S., Singh, S., Humblet-Baron, S., Ochs, H.D., Miao, C.H., and Rawlings, D.J. (2012). Ubiquitous high-level gene expression in hematopoietic lineages provides effective lentiviral gene therapy of murine Wiskott-Aldrich syndrome. Blood *119*, 4395–4407.

51. Cartier, N., Hacein-Bey-Abina, S., Von Kalle, C., Bougnères, P., Fischer, A., Cavazzana-Calvo, M., and Aubourg, P. (2010). [Gene therapy of x-linked adrenoleukodystrophy using hematopoietic stem cells and a lentiviral vector]. Bull. Acad. Natl. Med. *194*, 255–264. discussion 264-258.

52. Tsien, R.Y. (1998). The green fluorescent protein. Annu. Rev. Biochem. *67*, 509–544.

53. Donnelly, M.L.L., Luke, G., Mehrotra, A., Li, X., Hughes, L.E., Gani, D., and Ryan, M.D. (2001). Analysis of the aphthovirus 2A/2B polyprotein 'cleavage' mechanism indicates not a proteolytic reaction, but a novel translational effect: a putative ribosomal 'skip. J. Gen. Virol. *82*, 1013–1025.

54. Ozog, S., Timberlake, N.D., Hermann, K., Garijo, O., Haworth, K.G., Shi, G., Glinkerman, C.M., Schefter, L.E., D'Souza, S., Simpson, E., et al. (2019). Resveratrol trimer enhances gene delivery to hematopoietic stem cells by reducing antiviral restriction at endosomes. Blood *134*, 1298–1311.

55. Heffner, G.C., Bonner, M., Christiansen, L., Pierciey, F.J., Campbell, D., Smurnyy, Y., Zhang, W., Hamel, A., Shaw, S., Lewis, G., et al. (2018). Prostaglandin E(2) increases lentiviral vector transduction efficiency of adult human hematopoietic stem and progenitor cells. Mol. Ther. *26*, 320–328.

56. Bender, J.G., Unverzagt, K., Walker, D.E., Lee, W., Smith, S., Williams, S., and Van Epps, D.E. (1994). Phenotypic analysis and characterization of CD34+ cells from normal human bone marrow, cord blood, peripheral blood, and mobilized peripheral blood from patients undergoing autologous stem cell transplantation. Clin. Immunol. Immunopathol. *70*.

57. McKercher, S.R., Torbett, B.E., Anderson, K.L., Henkel, G.W., Vestal, D.J., Baribault, H., Klemsz, M., Feeney, A.J., Wu, G.E., Paige, C.J., et al. (1996). Targeted disruption of the PU.1 gene results in multiple hematopoietic abnormalities. EMBO J. *15*, 5647–5658.

58. Omori, S.A., and Wall, R. (1993). Multiple motifs regulate the B-cell-specific promoter of the B29 gene. Proc. Natl. Acad. Sci. USA *90*, 11723–11727. https://doi.org/10.1073/pnas.90.24.11723.

59. Hung, K.L., Meitlis, I., Hale, M., Chen, C.-Y., Singh, S., Jackson, S.W., Miao, C.H., Khan, I.F., Rawlings, D.J., and James, R.G. (2018). Engineering protein-secreting plasma cells by homology-directed repair in primary human B cells. Mol. Ther. *26*, 456–467.

60. Vamva, E., Ozog, S., Verhoeyen, E., James, R.G., Rawlings, D.J., and Torbett, B.E. (2022). An optimized measles virus glycoprotein-pseudotyped lentiviral vector production system to promote efficient transduction of human primary B cells. STAR Protoc *3*, 101228.

61. Zhao, Y., Stepto, H., and Schneider, C.K. (2017). Development of the first world health organization lentiviral vector standard: toward the production control and standardization of lentivirus-based gene therapy products. Hum. Gene Ther. Methods *28*, 205–214.

62. Davis-Gardner, M.E., Gardner, M.R., Alfant, B., and Farzan, M. (2017). eCD4-Ig promotes ADCC activity of sera from HIV-1-infected patients. PLoS Pathog. *13*, e1006786.

63. Liu, H., Saxena, A., Sidhu, S.S., and Wu, D. (2017). Fc engineering for developing therapeutic bispecific antibodies and novel scaffolds. Front. Immunol. *8*, 38.

64. Ridgway, J.B., Presta, L.G., and Carter, P. (1996). 'Knobs-into-holes' engineering of antibody CH3 domains for heavy chain heterodimerization. Protein Eng. *9*, 617–621.

65. Moore, G.L., Bautista, C., Pong, E., Nguyen, D.H., Jacinto, J., Eivazi, A., Muchhal, U.S., Karki, S., Chu, S.Y., and Lazar, G.A. (2011). A novel bispecific antibody format enables simultaneous bivalent and monovalent co-engagement of distinct target antigens. mAbs *3*, 546–557.

66. Klein, C., Sustmann, C., Thomas, M., Stubenrauch, K., Croasdale, R., Schanzer, J., Brinkmann, U., Kettenberger, H., Regula, J.T., and Schaefer, W. (2012). Progress in overcoming the chain association issue in bispecific heterodimeric IgG antibodies. mAbs *4*, 653–663.

67. Leaman, D.P., Lee, J.H., Ward, A.B., and Zwick, M.B. (2015). Immunogenic display of purified chemically cross-linked HIV-1 spikes. J. Virol. *89*, 6725–6745.

68. Choe, H., Li, W., Wright, P.L., Vasilieva, N., Venturi, M., Huang, C.C., Grundner, C., Dorfman, T., Zwick, M.B., Wang, L., et al. (2003). Tyrosine sulfation of human antibodies contributes to recognition of the CCR5 binding region of HIV-1 gp120. Cell *114*, 161–170.

69. Chan, J.T.H., Liu, Y., Khan, S., St-Germain, J.R., Zou, C., Leung, L.Y.T., Yang, J., Shi, M., Grunebaum, E., Campisi, P., et al. (2018). A tyrosine sulfation-dependent HLA-I modification identifies memory B cells and plasma cells. Sci. Adv. *4*, eaar7653.

70. Sasaki, N., Hosoda, Y., Nagata, A., Ding, M., Cheng, J.M., Miyamoto, T., Okano, S., Asano, A., Miyoshi, I., and Agui, T. (2007). A mutation in Tpst2 encoding tyrosylprotein sulfotransferase causes dwarfism associated with hypothyroidism. Mol. Endocrinol. *21*, 1713–1721.

71. Stone, M.J., Chuang, S., Hou, X., Shoham, M., and Zhu, J.Z. (2009). Tyrosine sulfation: an increasingly recognised post-translational modification of secreted proteins. N Biotechnol. *25*, 299–317.

72. Klijn, C., Durinck, S., Stawiski, E.W., Haverty, P.M., Jiang, Z., Liu, H., Degenhardt, J., Mayba, O., Gnad, F., Liu, J., et al. (2015). A comprehensive transcriptional portrait of human cancer cell lines. Nat. Biotechnol. *33*, 306–312.

73. Huang, D., Tran, J.T., Olson, A., Vollbrecht, T., Tenuta, M., Guryleva, M.V., Fuller, R.P., Schiffner, T., Abadejos, J.R., Couvrette, L., et al. (2020). Vaccine elicitation of HIV broadly neutralizing antibodies from engineered B cells. Nat. Commun. *11*, 5850.

74. Han, J., Tam, K., Ma, F., Tam, C., Aleshe, B., Wang, X., Quintos, J.P., Morselli, M., Pellegrini, M., Hollis, R.P., and Kohn, D.B. (2021). β-Globin lentiviral vectors have reduced titers due to incomplete vector RNA genomes and lowered virion production. Stem Cell Rep. *16*, 198–211.

75. Kumar, M., Keller, B., Makalou, N., and Sutton, R.E. (2001). Systematic determination of the packaging limit of lentiviral vectors. Hum. Gene Ther. *12*, 1893–1905.

76. Wang, Q., Chen, Y., Park, J., Liu, X., Hu, Y., Wang, T., McFarland, K., and Betenbaugh, M.J. (2019). Design and production of bispecific antibodies. Antibodies *8*.

77. Traxlmayr, M.W., Hasenhindl, C., Hackl, M., Stadlmayr, G., Rybka, J.D., Borth, N., Grillari, J., Ruker, F., and Obinger, C. (2012). Construction of a stability landscape of the CH3 domain of human IgG1 by combining directed evolution with high throughput sequencing. J. Mol. Biol. *423*, 397–412.

78. Merchant, A.M., Zhu, Z., Yuan, J.Q., Goddard, A., Adams, C.W., Presta, L.G., and Carter, P. (1998). An efficient route to human bispecific IgG. Nat. Biotechnol. *16*, 677–681.

79. Shields, R.L., Namenuk, A.K., Hong, K., Meng, Y.G., Rae, J., Briggs, J., Xie, D., Lai, J., Stadlen, A., Li, B., et al. (2001). High resolution mapping of the binding site on human IgG1 for Fc gamma RI, Fc gamma RII, Fc gamma RIII, and FcRn and design of IgG1 variants with improved binding to the Fc gamma R. J. Biol. Chem. *276*, 6591–6604.

80. Idusogie, E.E., Wong, P.Y., Presta, L.G., Gazzano-Santoro, H., Totpal, K., Ultsch, M., and Mulkerrin, M.G. (2001). Engineered antibodies with increased activity to recruit complement. J. Immunol. *166*, 2571–2575.

81. Niehrs, C., Beisswanger, R., and Huttner, W.B. (1994). Protein tyrosine sulfation, 1993–an update. Chem. Biol. Interact. *92*, 257–271.

82. Ludeman, J.P., and Stone, M.J. (2014). The structural role of receptor tyrosine sulfation in chemokine recognition. Br. J. Pharmacol. *171*, 1167–1179.

83. Bekker, L.G., Tatoud, R., Dabis, F., Feinberg, M., Kaleebu, P., Marovich, M., Ndung'u, T., Russell, N., Johnson, J., Luba, M., et al. (2020). The complex challenges of HIV vaccine development require renewed and expanded global commitment. Lancet *395*, 384–388.

84. Shirley, J.L., de Jong, Y.P., Terhorst, C., and Herzog, R.W. (2020). Immune responses to viral gene therapy vectors. Mol. Ther. *28*, 709–722.

85. Witmer, C., and Young, G. (2012). Factor VIII inhibitors in hemophilia A: rationale and latest evidence. Therapeutic Advances in Hematol. *4*, 59–72.

86. Moots, R.J., Xavier, R.M., Mok, C.C., Rahman, M.U., Tsai, W.-C., Al-Maini, M.H., Pavelka, K., Mahgoub, E., Kotak, S., Korth-Bradley, J., et al. (2017). The impact of anti-drug antibodies on drug concentrations and clinical outcomes in rheumatoid arthritis patients treated with adalimumab, etanercept, or infliximab: results from a multinational, real-world clinical practice, non-interventional study. PLoS One *12*.

87. Calcedo, R., Somanathan, S., Qin, Q., Betts, M.R., Rech, A.J., Vonderheide, R.H., Mueller, C., Flotte, T.R., and Wilson, J.M. (2017). Class I-restricted T-cell responses to a polymorphic peptide in a gene therapy clinical trial for α-1-antitrypsin deficiency. Proc. Natl. Acad. Sci. USA *114*, 1655–1659.

88. Mendell, J.R., Campbell, K., Rodino-Klapac, L., Sahenk, Z., Shilling, C., Lewis, S., Bowles, D., Gray, S., Li, C., Galloway, G., et al. (2010). Dystrophin immunity in Duchenne's muscular dystrophy. N. Engl. J. Med. *363*, 1429–1437.

89. Melo, M.E., Qian, J., El-Amine, M., Agarwal, R.K., Soukhareva, N., Kang, Y., and Scott, D.W. (2002). Gene transfer of Ig-fusion proteins into B cells prevents and treats autoimmune diseases. J. Immunol. *168*, 4788–4795.

90. Skupsky, J., Zhang, A.H., Su, Y., and Scott, D.W. (2010). B-cell-delivered gene therapy induces functional T regulatory cells and leads to a loss of antigen-specific effector cells. Mol. Ther. *18*, 1527–1535.

91. Lei, T.C., Su, Y., and Scott, D.W. (2005). Tolerance induction via a B-cell delivered gene therapy-based protocol: optimization and role of the Ig scaffold. Cell. Immunol. *235*, 12–20.

92. Soukhareva, N., Jiang, Y., and Scott, D.W. (2006). Treatment of diabetes in NOD mice by gene transfer of Ig-fusion proteins into B cells: role of T regulatory cells. Cell. Immunol. *240*, 41–46.

93. Frecha, C., Costa, C., Nègre, D., Gauthier, E., Russell, S.J., Cosset, F.L., and Verhoeyen, E. (2008). Stable transduction of quiescent T cells without induction of cycle progression by a novel lentiviral vector pseudotyped with measles virus glycoproteins. Blood *112*, 4843–4852.

94. Ozog, S., Chen, C.X., Simpson, E., Garijo, O., Timberlake, N.D., Minder, P., Verhoeyen, E., and Torbett, B.E. (2019). CD46 null packaging cell line improves measles lentiviral vector production and gene delivery to hematopoietic stem and progenitor cells. Mol. Ther. Methods Clin. Dev. *13*, 27–39.

95. Vamva, E., Lever, A.M.L., Vink, C.A., and Kenyon, J.C. (2020). Development of a novel competitive qRT-PCR assay to measure relative lentiviral packaging efficiency. Mol. Ther. Methods Clin. Dev. *19*, 307–319.

96. Mbisa, J.L., Delviks-Frankenberry, K.A., Thomas, J.A., Gorelick, R.J., and Pathak, V.K. (2009). Real-time PCR analysis of HIV-1 replication post-entry events. Methods Mol. Biol. *485*, 55–72.

97. Liao, H.X., Lynch, R., Zhou, T., Gao, F., Alam, S.M., Boyd, S.D., Fire, A.Z., Roskin, K.M., Schramm, C.A., Zhang, Z., et al. (2013). Co-evolution of a broadly neutralizing HIV-1 antibody and founder virus. Nature *496*, 469–476.

98. Leaman, D.P., Kinkead, H., and Zwick, M.B. (2010). In-solution virus capture assay helps deconstruct heterogeneous antibody recognition of human immunodeficiency virus type 1. J. Virol. *84*, 3382–3395.

99. Dobin, A., Davis, C.A., Schlesinger, F., Drenkow, J., Zaleski, C., Jha, S., Batut, P., Chaisson, M., and Gingeras, T.R. (2013). STAR: ultrafast universal RNA-seq aligner. Bioinformatics *29*, 15–21.

100. R Core Team (2020). R: A Language and Environment for Statistical Computing (R Foundation for Statistical Computing).

OMTM, Volume *28*

Supplemental information

A lentiviral vector B cell gene therapy platform for the delivery of the anti-HIV-1 eCD4-Ig-knob-in-hole-reversed immunoadhesin

Eirini Vamva, Stosh Ozog, Daniel P. Leaman, Rene Yu-Hong Cheng, Nicholas J. Irons, Andee Ott, Claire Stoffers, Iram Khan, Geraldine K.E. Goebrecht, Matthew R. Gardner, Michael Farzan, David J. Rawlings, Michael B. Zwick, Richard G. James, and Bruce E. Torbett

Supplemental Figures

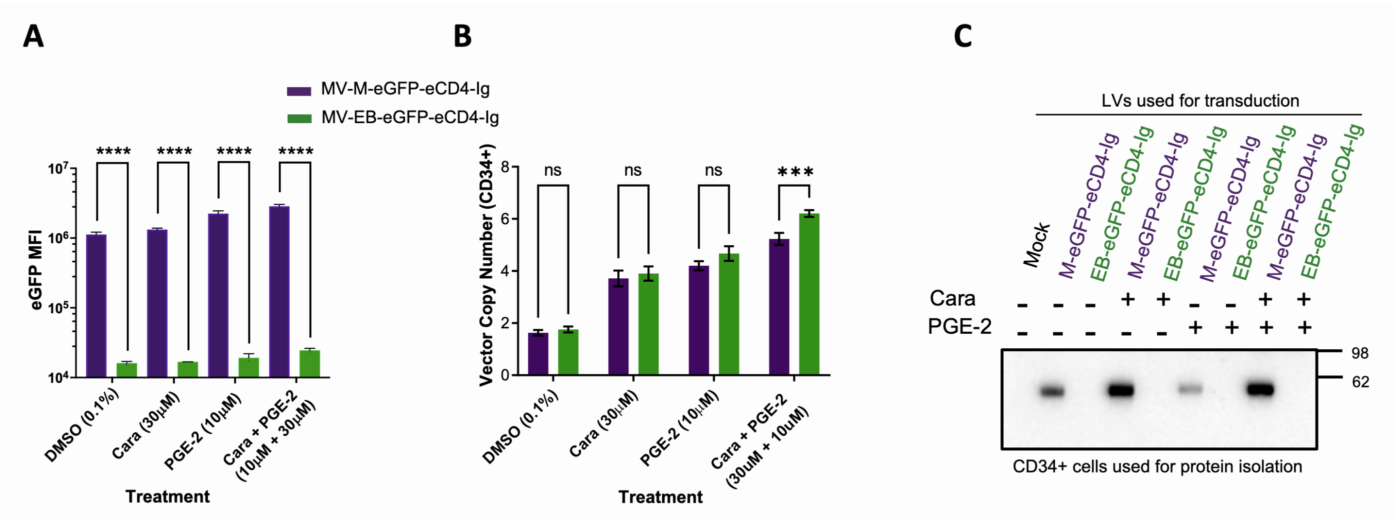

Figure S1. The B cell EμB29 promoter maintains low levels of eGFP expression in CD34$^+$ stem and progenitors without detectable eCD4-Ig in the culture supernatant as LV copy number increases. [A] Small molecule enhancement of LV M-eGFP-eCD4-Ig or EB-eGFP-eCD4-Ig transduction of umbilical cord blood CD34$^+$ stem and progenitors (HSPCs) and eGFP MFI detection 7 days post LV transduction. DMSO was the diluent for Cara and PGE-2. [B] Viral Copy Numbers (VCNs) from CD34$^+$ HSPCs evaluated in [A] for eGFP expression. [C] Assessment of eCD4-Ig production in culture supernatants as in Figure 1 [D]. Data presented in bar graphs are mean ± S.D. of triplicates. ns = not significant, ***p<0.001, ****p<0.0001.

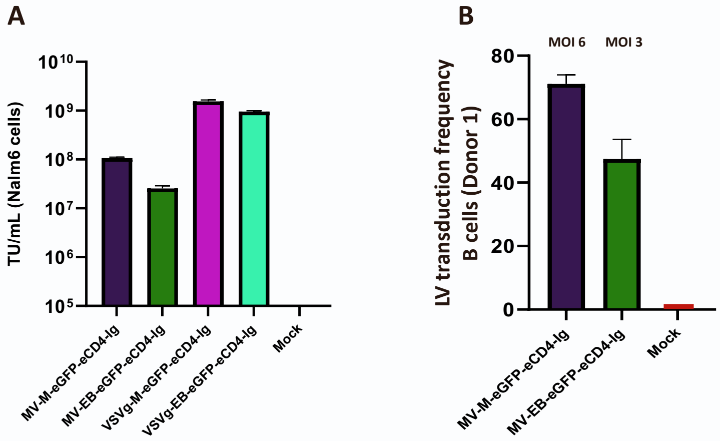

Figure S2. Measles pseudotyped (MV) and VSV-G LV transduction of Nalm6 B cells and MV LV transduction of primary B cells. [A] Titration of MV-M-eGFP-eCD4-Ig, MV-EB-eGFP-eCD4-Ig, VSV-G-M-eGFP-eCD4-Ig and VSV-G-MV-EB-eGFP-eCD4-Ig LVs by transduction of Nalm6 cells. Transduction units (TUs) were measured using flow cytometry five days post transduction (see Material and Methods for protocols). Transduction Units/mL (TU/mL) were calculated by flow cytometry of eGFP-expressing cells, using the dilution in which samples contained 4%–25% eGFP$^+$ cells. **[B]** 20 µL of MV-M-eGFP-eCD4-Ig (MOI 6) and MV-EB-eGFP-eCD4-Ig (MOI 3) were used to transduce primary B cells (Donor 1) as described in Materials and Methods. Transduction efficiency was measured by evaluating primary B cells for eGFP production using flow cytometry five days post LV transduction. Mock LV used the same conditions as LV transduction, but without the addition of LV. B cell Donor 1.

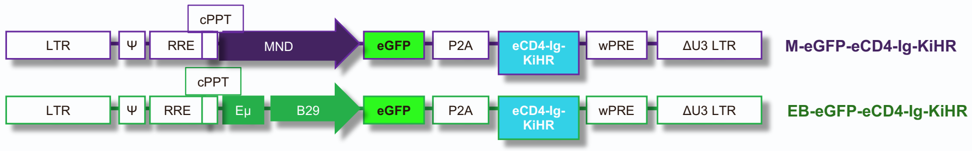

Figure S3. Diagram of LVs MND-eCD4-Ig-KiHR and EμB29-eCD4-Ig-KiHR. The diagram shows the inclusion of KiHR in eCD4-Ig (eCD4-Ig-KiHR) which includes the KiHR mutations F405A and T394F in the CH3 dimerization interface. See **Figure 3** for additional information on the CH3 mutations. Other LV elements remain the same as shown in **Figure 1B**.

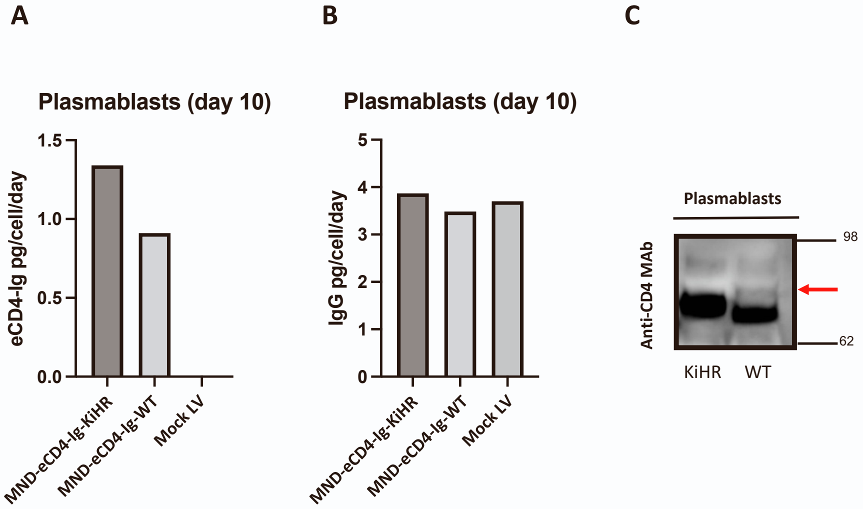

Figure S4. Production of eCD4-Ig-WT and eCD4-Ig-KiHR and IgG in LV transduced plasmablasts. [A] Measurements of eCD4-Ig and eCD4-Ig-KiHR and **[B]** IgG expression from plasmablast supernatants derived from primary B cells transduced with MV M-eGFP-eCD4-Ig-WT or MV M-eGFP-eCD4-Ig-KiHR LVs as described in Materials and Methods and **Figure 4**. IgG expression was measured with using an anti-IgG ELISA immunoassay and eCD4-Ig expression was quantified using an anti-gp140 ELISA assay. eCD4-Ig and IgG expression were normalized to the total number of live cells per day. **[C]** Detection of eCD4-Ig with the use of an anti-CD4 Mab non-reducing immunoblot. Red arrow identifies eCD4-Ig-WT and endogenous Ig Fc heterodimer association. B cells from PBMC of Donor 1.

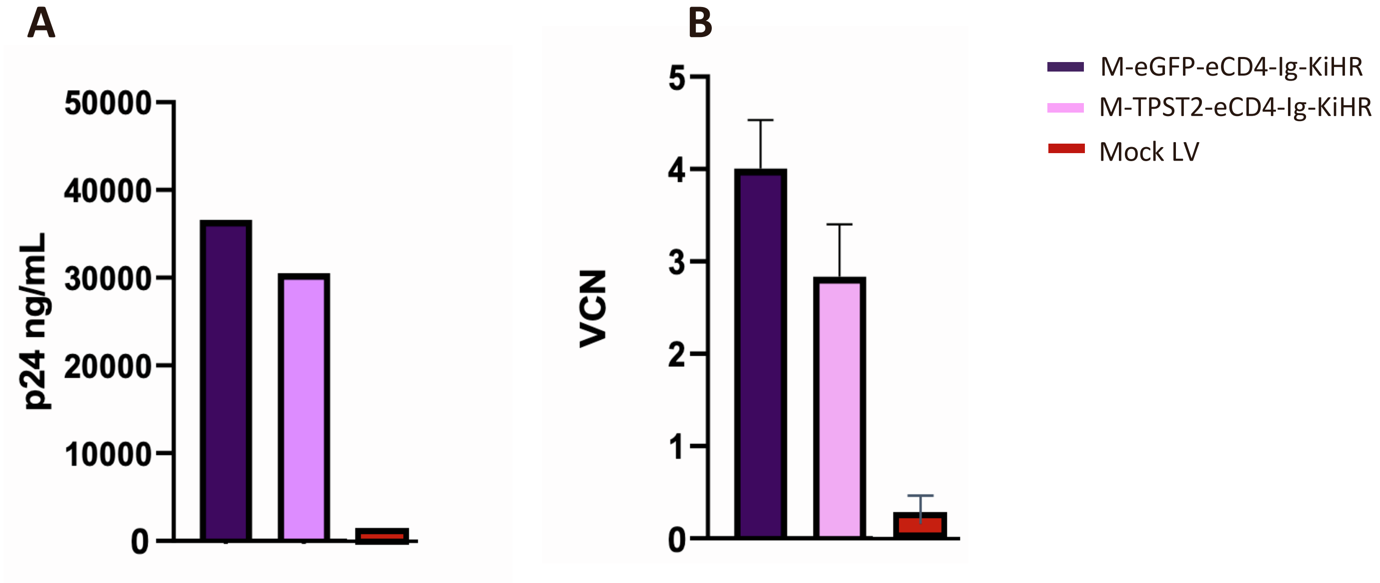

Figure S5. Characterization of MV M-eGFP-eCD4-Ig-KiHR and M-TPST2-eCD4-Ig-KiHR LV titers and B cell VCN after transduction. [A] ELISA p24 was performed on sucrose purified and concentrated MV M-eGFP-eCD4-Ig-KiHR and M-TPST2-eCD4-Ig-KiHR LV particles to measure physical titres. **[B]** MV-LVs were normalised for p24 content and used to transduce primary B cells (See **Figures 4, 5** for information on B donor and transduction protocols). B cell gDNA was extracted on the thirteenth day of the culture and samples were assessed for integrated provirus with digital droplet PCR (see Materials and Methods). Error bars show S.D. Wherever error bars are missing, it is due to lack of variability amongst duplicates.
